# Comparison of metabolic pathways of different α-*N*-heterocyclic thiosemicarbazones

**DOI:** 10.1007/s00216-018-0889-x

**Published:** 2018-02-23

**Authors:** Karla Pelivan, Lisa M. Frensemeier, Uwe Karst, Gunda Koellensperger, Petra Heffeter, Bernhard K. Keppler, Christian R. Kowol

**Affiliations:** 1 Institute of Inorganic Chemistry, Faculty of Chemistry, University of Vienna, Waehringer Strasse 42, 1090 Vienna, Austria; 2 Institute of Inorganic and Analytical Chemistry, University of Muenster, Corrensstrasse 28/30, 48149 Muenster, Germany; 3 Institute of Analytical Chemistry, Faculty of Chemistry, University of Vienna, Waehringer Strasse 38, 1090 Vienna, Austria; 4 Institute of Cancer Research and Comprehensive Cancer Center, Medical University of Vienna, Borschkegasse 8a, 1090 Vienna, Austria; 5 Research Cluster “Translational Cancer Therapy Research”, University and Medical University of Vienna, Vienna, Austria

**Keywords:** Drug metabolism, Thiosemicarbazones, Electrochemical oxidation, Microsomes, In vivo metabolism

## Abstract

Clinical failure of novel drugs is often related to their rapid metabolism and excretion. This highlights the importance of elucidation of their pharmacokinetic profile already at the preclinical stage of drug development. Triapine, the most prominent representative of α-*N*-heterocyclic thiosemicarbazones, was investigated in more than 30 clinical phase I/II trials, but the results against solid tumors were disappointing. Recent investigations from our group suggested that this is, at least partially, based on the fast metabolism and excretion. In order to establish more detailed structure/activity/metabolism relationships, herein a panel of 10 different Triapine derivatives was investigated for their metabolic pathways. From the biological point of view, the panel consists of terminally dimethylated thiosemicarbazones with nanomolar IC_50_ values, derivatives with micromolar cytotoxicities comparable to Triapine and a completely inactive representative. To study the oxidative metabolism, a purely instrumental approach based on electrochemistry/mass spectrometry was applied and the results were compared to the data obtained from microsomal incubations. Overall, the investigated thiosemicarbazones underwent the phase I metabolic reactions dehydrogenation, hydroxylation, oxidative desulfuration (to semicarbazone and amidrazone) and demethylation. Notably, dehydrogenation resulted in a ring-closure reaction with formation of thiadiazoles. Although strong differences between the metabolic pathways of the different thiosemicarbazones were observed, they could not be directly correlated to their cytotoxicities. Finally, the metabolic pathways for the most cytotoxic compound were elucidated also in tissues collected from drug-treated mice, confirming the data obtained by electrochemical oxidation and microsomes. In addition, the in vivo experiments revealed a very fast metabolism and excretion of the compound.

**Electronic supplementary material:**

The online version of this article (10.1007/s00216-018-0889-x) contains supplementary material, which is available to authorized users.

**Graphical abstract:**
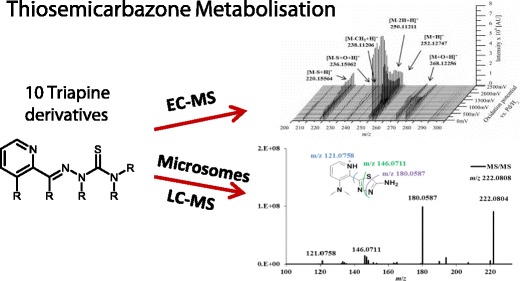
Structure/activity/metabolisation relationships for 10 anticancer thiosemicarbazones were established using electrochemical oxidation coupled to mass spectrometry (EC-MS) and human liver microsomes analyzed by LC-MS

## Introduction

A crucial part in drug development is the investigation of the pharmacokinetics and ADME (absorption, distribution, metabolism, excretion) profile of a new compound, as rapid metabolism and body clearance are common reasons behind the clinical failure of drugs [1, 2]. This highlights the importance of early metabolic studies in the drug optimization process in order to select the most promising candidate(s) for further evaluation [3]. In general, drug metabolism can be subdivided into two phases—modification (phase I) and conjugation (phase II) [4]. Phase I modification involves reactions such as oxidation, reduction, and hydroxylation of the compound [5]. Subsequently, the products can undergo phase II conjugation reactions (e.g., acetylation or glucuronidation) resulting in more hydrophilic metabolites, which can easily be excreted from the body [6].

**Scheme 1:**
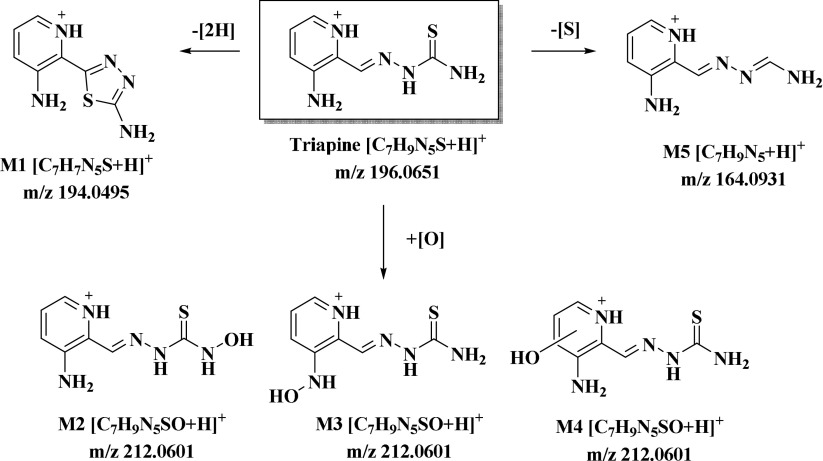
The metabolic pathways of Triapine [18]

The substance class of α-*N*-heterocyclic thiosemicarbazones has attracted high attention in the field of anticancer chemotherapy [7, 8]. With regard to the mode of action, the enzyme ribonucleotide reductase (RR), responsible for the reduction of ribonucleotides to the respective deoxyribonucleotides and, therefore, involved in DNA synthesis, has been identified as the principal molecular target [9, 10]. As this enzyme is frequently overexpressed in fast proliferating malignant tissues, it is a promising and well-known drug target [11]. Triapine (3-aminopyridine-2-carboxaldehyde thiosemicarbazone; Scheme 1), the most prominent representative among α-*N*-heterocyclic thiosemicarbazones, entered first clinical trials 15 years ago [12, 13]. Unfortunately, despite more than 30 phase I and II clinical trials, only activity against hematological diseases was observed, while this drug was widely ineffective against various solid tumors [13, 14]. Surprisingly, information on the ADME behavior of Triapine is rarely described in literature. Only an approximate half-life time of 1 h was reported in patients after *i.v.* administration [15], as well as very briefly its metabolism and excretion via acetylation and hydroxylation [16]. In line with these reports, our investigations on Triapine in mice showed also a short plasma half-life time and fast excretion [17, 18]. Furthermore, we recently studied the metabolic pathways of Triapine by the application of different analytical tools, including electrochemical oxidation, microsomal incubation and in vivo experiments [18]. The data showed that the key metabolites of Triapine are (1) the dehydrogenated ring-closed thiadiazole (M1, Scheme 1), which lost the crucial chemical property of anticancer α-*N*-heterocyclic thiosemicarbazones to coordinate biologically relevant metal ions and showed no cytotoxic activity and (2) different hydroxylated species (M2–M4, Scheme 1) [18]. Notably, already in case of 5-HP (5-hydroxypyridine-2-carboxaldehyde thiosemicarbazones; see Electronic Supplementary Material (ESM) Scheme S1), the first clinically investigated thiosemicarbazone, the drug metabolism was crucial, as a clinical phase I trial showed very rapid metabolism and excretion via glucuronidation (plasma half-life time < 10 min) [19, 20]. Since 2015, two new thiosemicarbazones Coti-2 ((*E*)-*N*′-(6,7-dihydroquinolin-8(5*H*)-ylidene)-4-(pyridin-2-yl)piperazine-1-carbothiohydrazide; ESM Scheme S1) and DpC (di-2-pyridylketone-4-cyclohexyl-4-methyl-3-thiosemicarbazone; ESM Scheme S1) are evaluated in clinical trials [21–23]. Regarding the biotransformation of DpC, the main metabolic reaction in vivo was reported to be an oxidative desulfuration with formation of an amidrazone [24]. This metabolite was observed only in very low levels in case of Triapine in our studies (Scheme 1), which already indicates strong differences between different thiosemicarbazone representatives.

**Scheme 2:**
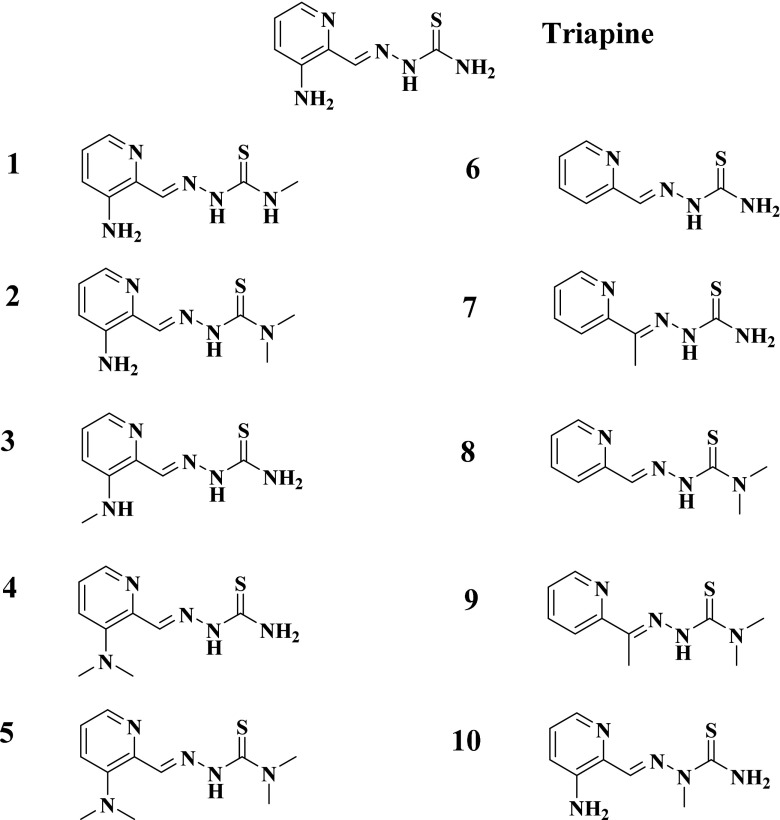
Molecular structures of the investigated α-*N*-heterocyclic thiosemicarbazones (**1**–**10**) and Triapine

The aim of the work presented here was to investigate the influence of the presence/absence as well as the position of different functional groups on the metabolic behavior of α-*N*-heterocyclic thiosemicarbazones. To this end, the metabolites of 10 different Triapine derivatives (Scheme 2) were investigated. We selected derivatives with “step-by-step” chemical modifications compared to the reference compound Triapine. Moreover, these thiosemicarbazones cover a broad spectrum of anticancer activity from IC_50_ values in the low nanomolar range up to > 100 μM. All compounds were analyzed by the application of electrochemistry and microsomal incubations, with subsequent detection of the metabolites via an optimized LC-HRMS method. Finally, for one of the nanomolar cytotoxic compounds, the in vivo metabolic behavior was evaluated as well. The obtained data were analyzed regarding structure/metabolism relationships and related to the metabolic pathways observed for Triapine.

## Materials and methods

### Chemicals

Thiosemicarbazones **1**–**10** were synthesized according to literature procedures [25–27]. Human liver microsomes were obtained from BD Gentest (Woburn, MA, USA), nicotinamide adenine dinucleotide phosphate (NADPH) was purchased from Roche Diagnostics (Mannheim, Germany) and phosphate-buffered saline (PBS, pH 7.4, 10×) from Gibco^®^ by Life Technologies™ (Carlsbad, CA, USA). Formic acid (99–100%), ammonium formate (≥ 99.995% trace metals basis), LC-MS grade water and acetonitrile, as well as all other chemicals, were purchased from Sigma Aldrich, Austria.

### EC/ESI-HRMS measurements

For metabolism simulation, the electrochemical oxidation of the investigated thiosemicarbazones was carried out using a FlexCell (Antec Scientific, Zoeterwoude, The Netherlands) consisted of a three-electrode arrangement; a boron-doped diamond (BDD) working electrode, a graphite-doped teflon auxiliary electrode and a Pd/H_2_ reference electrode. For recording mass voltammograms, a homemade potentiostat was applied for the oxidation potential ramp from 0 and + 2500 mV (vs. Pd/H_2_) with a scan rate of 10 mV/s. The respective solution of the investigated compound (10 μmol/L) was prepared in 10 mM aqueous ammonium formate (adjusted to pH 7.4) and acetonitrile (50/50, *v*/*v*). A continuous flow rate of 10 μL/min was applied for pumping the compound solution through the electrochemical cell. The EC cell effluent was coupled online via an electrospray ionization (ESI) source to an Exactive™ HRMS from Thermo Fisher Scientific (Bremen, Germany). Full scan spectra (*m*/*z* 100–500 or *m*/*z* 100–600) were recorded in positive ionization mode at a resolution of 50,000 using the following conditions: sheath gas flow rate 10 a.u., auxiliary and sweep gas flow rate 0.0 a.u., spray voltage 4.0 kV, capillary temperature 280 °C, capillary voltage 30.0 V, tube lens voltage 55.0 V, skimmer voltage 16.0 V. XCalibur 2.1 software (Thermo Fisher Scientific, Bremen, Germany) was used for instrument control and data processing. The software Origin 9.1 (OriginLab, Northhampton, MA, USA) was used for data visualization.

### LC/ESI-HRMS measurements

A detailed investigation of the electrochemically generated metabolites was achieved with an optimized separation method, established by means of reversed phase liquid chromatography (RP-LC) coupled to ESI-HRMS. Prior to injection, the respective thiosemicarbazone solution (10 μmol/L) was oxidized at a constant potential of + 1800 mV vs. Pd/H_2_. The system was equipped with the Vanquish™ UHPLC coupled to a Q Exactive™ HF hybrid quadrupole-Orbitrap mass spectrometer and was controlled by XCalibur 2.1 software (Thermo Fisher Scientific, Bremen, Germany). An Atlantis T3 C18 reversed-phase column (150 mm × 2.1 mm, 3 μm particle size) from Waters (Milford, USA) was used for chromatographic separation. A water solution containing 1% acetonitrile and 0.1% formic acid was used as eluent A and acetonitrile solution containing 1% water and 0.1% formic acid as eluent B. The gradient elution was conducted with a flow rate of 200 μL/min with a following program: 0–1 min 1% B, 1–15 min 99% B, 15–16 min 99% B, 16–16.1 min 1% B, 16.1–23 min 1% B. The oxidized solutions were analyzed via LC/ESI-HRMS runs in positive ionization mode at a resolution of 120,000. The following optimized parameters were applied: injection volume 2 μl, column temperature 25 °C, autosampler temperature 5 °C, HESI source 320 °C, sheath gas flow rate 40 a.u., auxiliary gas flow rate 10 a.u., sweep gas flow rate 2 a.u., spray voltage 3.5 kV, capillary temp 300 °C, capillary voltage 35.0 V, tube lens voltage 55.0 V, skimmer voltage 16.0 V and a full scan MS from *m*/*z* 100–900. MS/MS experiments using higher-energy collisional dissociation (HCD) were performed for structural elucidation of the metabolites.

### Metabolisation investigations with human liver microsomes (HLM)

Metabolic pathways of the investigated thiosemicarbazones were elucidated by incubation with HLM as reported previously [28]. The incubation solutions consisted of the respective thiosemicarbazone (40 μM), HLM (1.3 mg/mL) and the co-factor NADPH (2.5 mM) in 50 mM phosphate buffer (pH 7.4) containing 5 mM MgCl_2_. Further solutions involved a substrate blank (without the drug), a co-factor blank (without NADPH) an enzyme blank (without HLM) and the positive control (Amodiaquine [29] instead of the thiosemicarbazone drug). All samples were incubated for 2 h at 37 °C. The metabolic reactions were stopped by the addition of ice-cold acetonitrile containing 0.1% formic acid (250 μL), followed by centrifugation at 17,000*g* (4 °C for 15 min) in order to remove the (protein) precipitate. Finally, the samples were subjected to the previously described LC/ESI-HRMS analysis (see above for LC/ESI-HRMS measurements).

### Animals

Six- to eight-week-old Balb/c mice were purchased from Harlan (Italy) and were housed under standard conditions with a 12-h light-dark cycle at the animal research facility with ad libitum access to food and water. The experiments were performed according to the Federation of Laboratory Animal Science Association guidelines for the use of experimental animals and were approved by the Ethics Committee for the Care and Use of Laboratory Animals at the Medical University Vienna and the Ministry of Science and Research, Austria (BMWF-66.009/0084-II/3b/2013). With regard to the execution of our animal experiments, we followed the ARRIVE guidelines.

### In vivo experiments

For investigation of pharmacokinetic behavior of **5** in vivo, the mice were treated with one intravenous injection of **5** (5 mg kg^−1^ dissolved in 10% propyleneglycol, 0.9% NaCl). After 15 min animals were anesthetized by Rompun©/Ketavet© and the organs (kidney, liver) were collected and stored at − 80 °C. Urine and blood were collected by bladder and heart puncture, respectively. At room temperature, blood was allowed to clot for 15–20 min. Serum was isolated by twofold centrifugation at 1800*g* for 10 min and stored at − 80 °C. The drug-free samples of untreated mice were collected for control using the same protocol.

### Sample preparation and measurements of the in vivo samples

Prior to the drug distribution analysis, the sample preparation protocol was applied on the collected in vivo samples. To this end, the tissues kidney and liver were homogenized in phosphate-buffered saline (1:3) in Micro packaging vials (2 mL; Peqlab, Erlangen, Germany) with Precellys ceramic beads (2.8 mm; Peqlab, Erlangen, Germany) for 3 × 10 s at 6000*g* with Minilys homogenizer (Bertin Technologies, Versailles, France) for the extraction. The so prepared liver and kidney extracts, together with collected mice serum and urine, were diluted 1:3 with acetonitrile, shaken vigorously. After centrifugation (10 min at 6000*g*) and dilution with water (1:2), the samples were measured via LC/ESI-HRMS (see above for the details of the LC/ESI-HRMS measurements).

## Results

### Compound panel selection

The first set of the compound panel (**1**–**5**) includes direct Triapine derivatives with one, two or four additional methyl groups on one or both of the NH_2_-moieties (Scheme 2). Additionally, to evaluate the effect of the amino group at the pyridine ring, the 2-formylpyridine (**6**) analogue was included together with the 2-acetylpyridine (**7**) derivative with a methyl group instead of the hydrogen at the C=N bond. In addition, derivatives of compound **6** and **7** with *N*-terminal dimethylation were selected (compound **8** and **9**). Finally, a direct Triapine derivative with a methylation at the hydrazinic NH-moiety was chosen as compound **10**. These structural modifications on the Triapine backbone also cause distinct differences in their biological activity. Our reference compound Triapine revealed a cytotoxic activity (IC_50_ values) of approximately 0.5 μM in different cancer cell lines [25–27]. The mono- and di-*N*-methylated derivatives **1**–**4** showed cytotoxic values comparable to that of Triapine (IC_50_ ~ 0.3–6 μM), whereas for the completely *N*-methylated derivative **5** a ~ 100-fold increase in the cytotoxicity was observed (IC_50_ 0.060–0.007 μM depending on the cell line) [27]. The obtained antiproliferative activities of **6** and **7** are comparable to Triapine (both ~ 3 μM), whereas **8** and **9** possess again a strongly increased cytotoxic activity in the nanomolar range [25, 26]. These data clearly show that a lack of any NH_2_ group is essential for nanomolar cytotoxicity. On the contrary, methylation of the hydrazinic-NH (compound **10)** resulted in a complete inactivation of Triapine with an IC_50_ value > 100 μM (unpublished data).

### Electrochemical oxidation of thiosemicarbazones

**Fig. 1:**
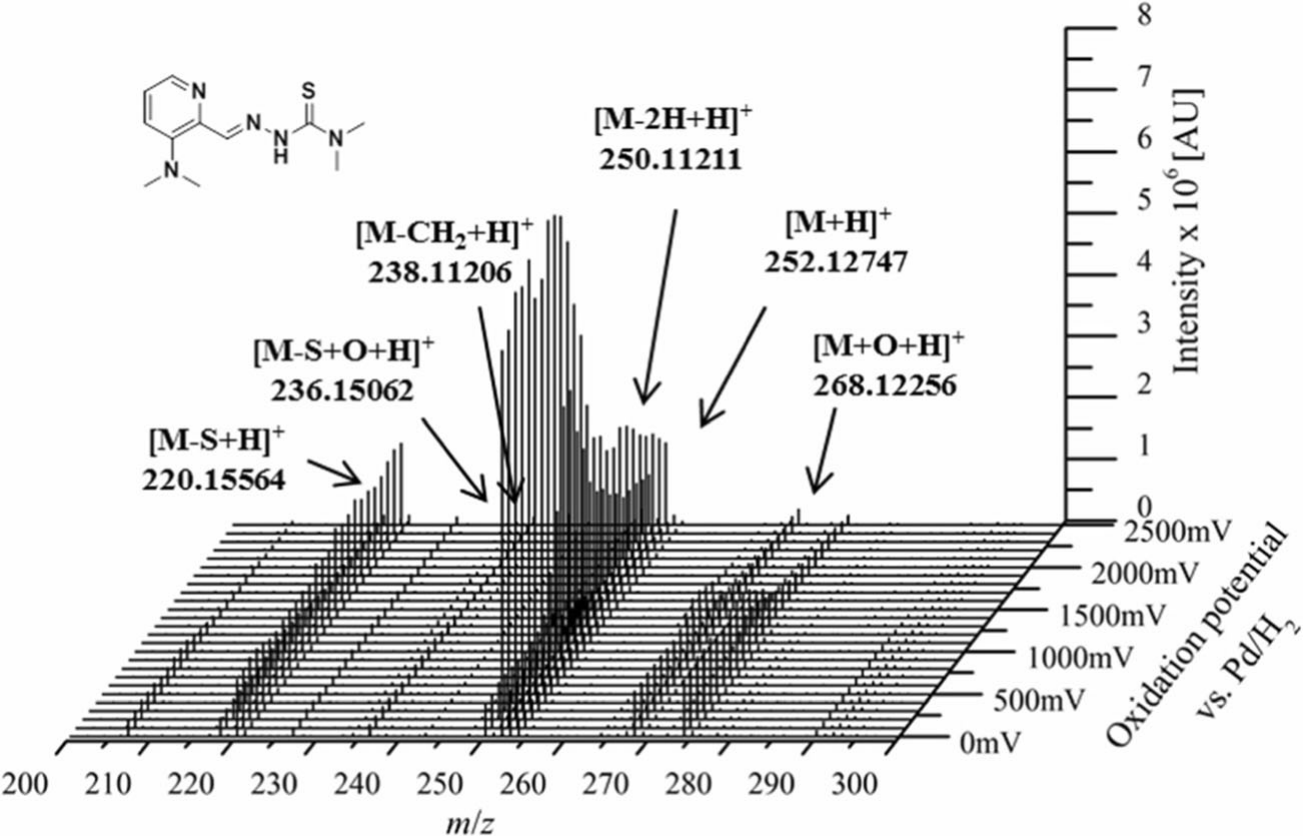
Mass voltammogram of thiosemicarbazone **5**

**Fig. 2:**
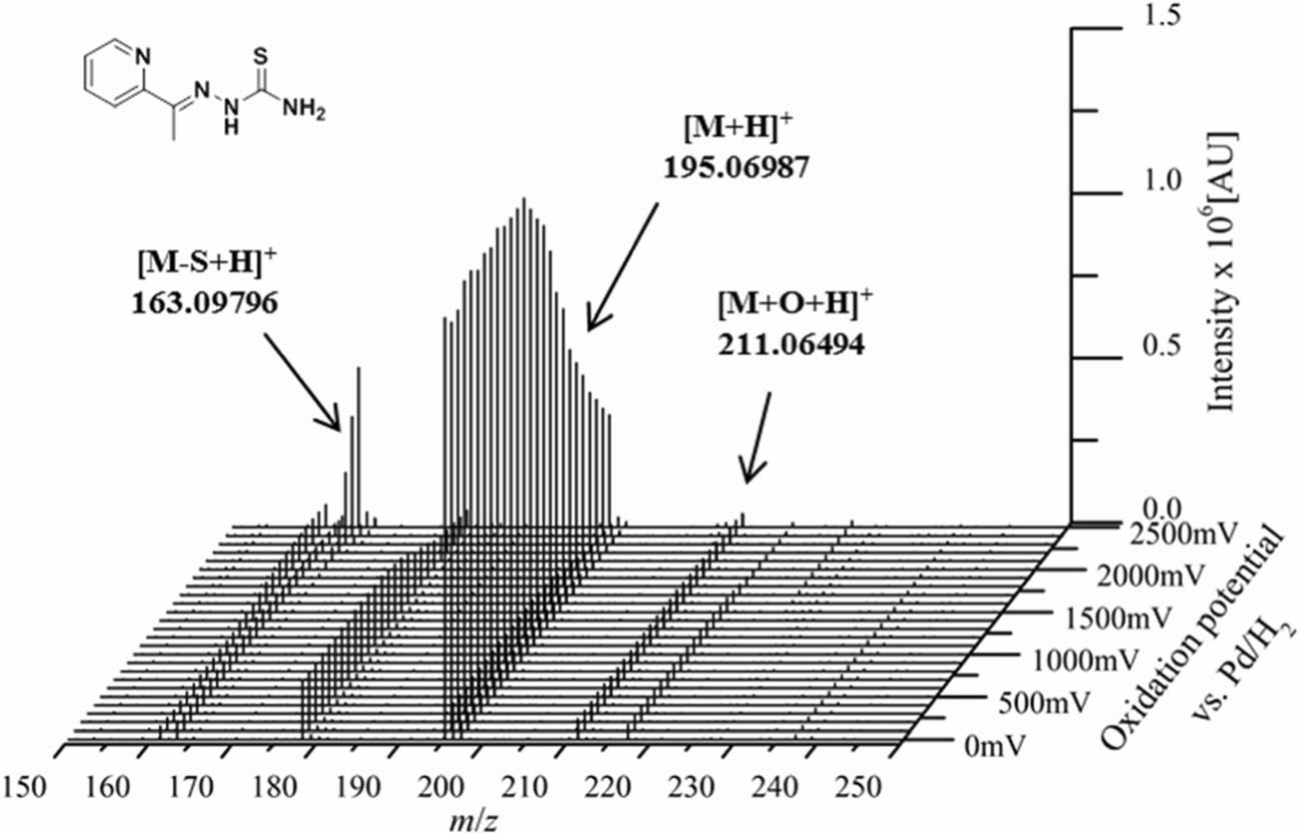
Mass voltammogram of thiosemicarbazone **7**

In the first step, the metabolic conversion of the thiosemicarbazones was investigated using electrochemistry, coupled to high resolution mass spectrometer (HRMS), a purely instrumental approach used for simulation of many oxidative liver reactions [30–32]. To this end, a potential (0 to + 2500 mV vs. Pd/H_2_, 10 mV/s) was applied to an electrochemical cell, through which the respective drug dissolved in aqueous ammonium formate pH 7.4/acetonitrile (1:1 *v*/*v*) was continuously pumped. The oxidized solution was finally introduced into an ESI-HRMS for the detection of the obtained products. By plotting the mass spectra against the applied potential ramp, three-dimensional mass voltammograms of the investigated drugs were generated (Figs. 1 and 2). The compounds were detected as [M + H]^+^ ions and (except compound **10**) upon interface degradation [M–NR_2_]^+^ as a thiocarbonyl ion [RC=S]^+^. At higher voltages, an intensity decay of the respective compound was observed simultaneously with the formation of several metabolites (Fig. 1). From the HRMS data, the sum formulae were calculated, which allowed the identification of the detected oxidation products (mass deviations between the calculated and theoretical *m*/*z* ratios of the obtained metabolites were always ≤ 3 ppm).

In general, for all the investigated thiosemicarbazones, the identified products of the metabolic reactions were hydroxylation [M + O + H]^+^, oxidative desulfuration (formation of the amidrazone and for some derivatives also the semicarbazone) and disulfide dimer formation [2M–2H + H]^+^. Dehydrogenation [M–2H + H]^+^ was observed for all thiosemicarbazones with exception of the 2-acetylpyridine derivatives **7** and **9**. In our previous work with Triapine, the dehydrogenation resulted in a ring-closure reaction with formation of a thiadiazole [18]. In case of **7** and **9**, this thiadiazole metabolite cannot be formed due to the methyl group at the C=N carbon atom instead of the hydrogen. Finally, demethylation [M–CH_2_ + H]^+^ was found to occur, however, only in case of **4** and **5**, although all the investigated compounds (except **6**) are thiosemicarbazones with at least one methyl group.

As examples of electrochemical oxidation and the obtained mass voltammograms the metabolic conversions of **5** and **7** are depicted in Figs. 1 and 2. In case of **5** ([M + H]^+^ *m/z* 252.1277), the main metabolic reactions were dehydrogenation ([M–2H + H]^+^ *m/z* 250.1121), hydroxylation ([M + O + H]^+^ *m*/*z* 268.1227), oxidative desulfuration (to both semicarbazone [M–S + O + H]^+^ *m/z* 236.1506 and amidrazone [M–S + H]^+^ *m/z* 220.1557), demethylation ([M–CH_2_ + H]^+^ *m/z* 238.1121) and disulfide formation ([2 M–2H + H]^+^ *m*/*z* 501.2326; not shown in Fig. 1). In traces, also the subsequent dehydrogenation of the metabolites (obtained after hydroxylation, demethylation, oxidative desulfuration and disulfide formation) was observed. All metabolites of **5** obtained by means of EC/MS are listed in Table 1. On the contrary, the electrochemical oxidation and the generated mass voltammogram of **7** ([M + H]^+^ *m/z* 195.0699) revealed only hydroxylation ([M + O + H]^+^ *m/z* 211.0648), oxidative desulfuration (to amidrazone [M–S + H]^+^ *m/z* 163.0978) and disulfide dimer formation reactions ([2M–2H + H]^+^ *m*/*z* 387.1169; not shown in Fig. 2). For the formed amidrazone and hydroxylated products also the subsequent dehydrogenation step was observed. All conversion products are listed in Table 2. The data of these two thiosemicarbazones already indicated some distinct differences in the metabolisation of diverse derivatives. An overview of all mass voltammograms and oxidation products identified for the other eight investigated thiosemicarbazones is given in Figs. S1-S8 and Tables S1-S8 in the ESM.

**Table 1:** Metabolites of compound **5** detected by EC/MS

Modification	Sum formula	Detected *m*/*z*	Theoretical *m*/*z*	Rel. deviation [ppm]	Metabolic reaction
[M + H]^+^	C_11_H_18_N_5_S	252.12747	252.12774	1.08	–
[M–N–2xCH_3_]^+^	C_9_H_11_N_4_S	207.06998	207.06989	0.42	
[M–2H + H]^+^	C_11_H_16_N_5_S	250.11211	250.11209	0.07	Dehydrogenation
[M–4H + H]^+^	C_11_H_14_N_5_S	248.09650	248.09644	0.23	Dehydrogenation
[M + O + H]^+^	C_11_H_18_N_5_SO	268.12256	268.12266	0.36	Hydroxylation
[M + O–2H + H]^+^	C_11_H_16_N_5_SO	266.10681	266.10701	0.74	Hydroxylation/ Dehydrogenation
[M + O–4H + H]^+^	C_11_H_14_N_5_SO	264.09116	264.09136	0.75	Hydroxylation/ Dehydrogenation
[M–S + O + H]^+^	C_11_H_18_N_5_O	236.15062	236.15059	0.14	Desulfuration
[M–S + O–2H + H]^+^	C_11_H_16_N_5_O	234.13492	234.13494	0.07	Desulfuration/ Dehydrogenation
[M–S + H]^+^	C_11_H_18_N_5_	220.15564	220.15567	0.15	Desulfuration
[M–S–2H + H]^+^	C_11_H_16_N_5_	218.14020	218.14002	0.82	Desulfuration/ Dehydrogenation
[M–CH_2_ + H]^+^	C_10_H_16_N_5_S	238.11206	238.11209	0.14	Demethylation
[M–CH_2_–2H + H]^+^	C_10_H_14_N_5_S	236.09641	236.09644	0.14	Demethylation/ Dehydrogenation
[M–2xCH_2_ + H]^+^	C_9_H_14_N_5_S	224.09621	224.09644	1.04	Demethylation*
[M–2xCH_2_–2H + H]^+^	C_9_H_12_N_5_S	222.08055	222.08079	1.09	Demethylation/ Dehydrogenation*
[M–3xCH_2_–2H + H]^+^	C_8_H_10_N_5_S	208.06487	208.06514	1.31	Demethylation/ Dehydrogenation*
[2M–2H + H]^+^	C_22_H_33_N_10_S_2_	501.23254	501.23256	0.04	Disulfide formation
[2M–4H + H]^+^	C_22_H_31_N_10_S_2_	499.21692	499.21691	0.02	Disulfide formation/ Dehydrogenation

*Observed after electrochemical oxidation only via LC-HRMS, but not via EC-HRMS

**Table 2:** Metabolites of compound **7** detected by EC/MS

Modification	Sum formula	Detected *m*/*z*	Theoretical *m*/*z*	Rel. deviation [ppm]	Metabolic reaction
[M + H]^+^	C_8_H_11_N_4_S	195.06987	195.06989	0.12	–
[M–NH_2_]^+^	C_8_H_8_N_3_S	178.04340	178.04334	0.31	
[M + O + H]^+^	C_8_H_11_N_4_SO	211.06494	211.06481	0.62	Hydroxylation
[M + O–2H + H]^+^	C_8_H_9_N_4_SO	209.04904	209.04916	0.57	Hydroxylation/ Dehydrogenation
[M–S + H]^+^	C_8_H_11_N_4_	163.09796	163.09782	0.84	Desulfuration
[M–S–2H + H]^+^	C_8_H_9_N_4_	161.08232	161.08217	0.91	Desulfuration/ Dehydrogenation
[M–CH_2_–2H + H]^+^	C_7_H_7_N_4_S	179.03848	179.03859	0.63	Demethylation/ Dehydrogenation*
[2M–2H + H]^+^	C_16_H_19_N_8_S_2_	387.11694	387.11686	0.21	Disulfide formation

*Observed after electrochemical oxidation only via LC-HRMS, but not via EC-HRMS

As a next step for all compounds also EC-LC-MS measurements were performed for a detailed analysis and detection of isomers. To this end, a reversed-phase liquid chromatographic separation (RP-LC) coupled to ESI-HRMS was developed. Prior to separation, all compounds **1**–**10** were electrochemically oxidized at a constant potential which was, equally to our studies with Triapine, set to + 1800 mV vs. Pd/H_2_ and subjected to the LC-HRMS.

**Fig. 3:**
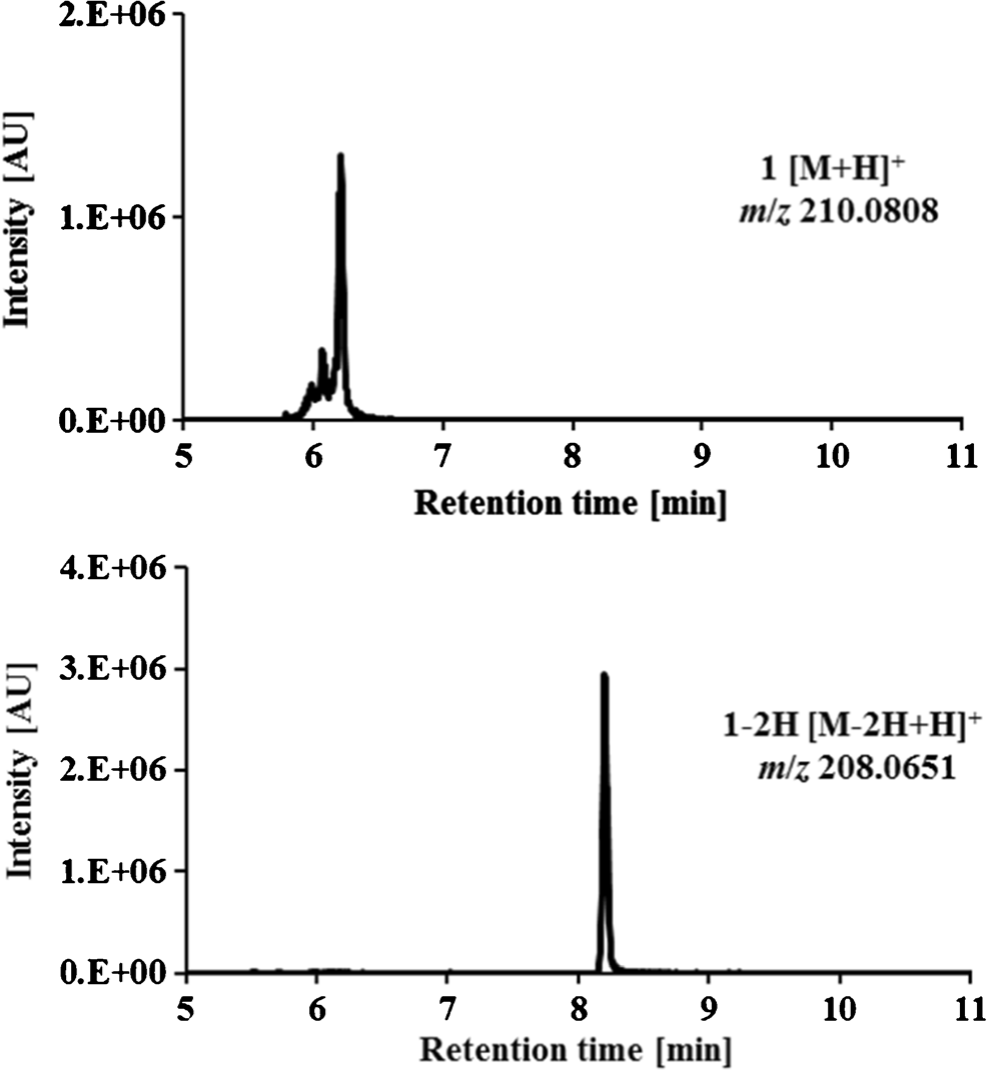
LC-HRMS analysis of terminally monomethylated Triapine (**1**) and its main dehydrogenated metabolite after electrochemical oxidation at + 1800 mV vs. Pd/H_2_

**Fig. 4:**
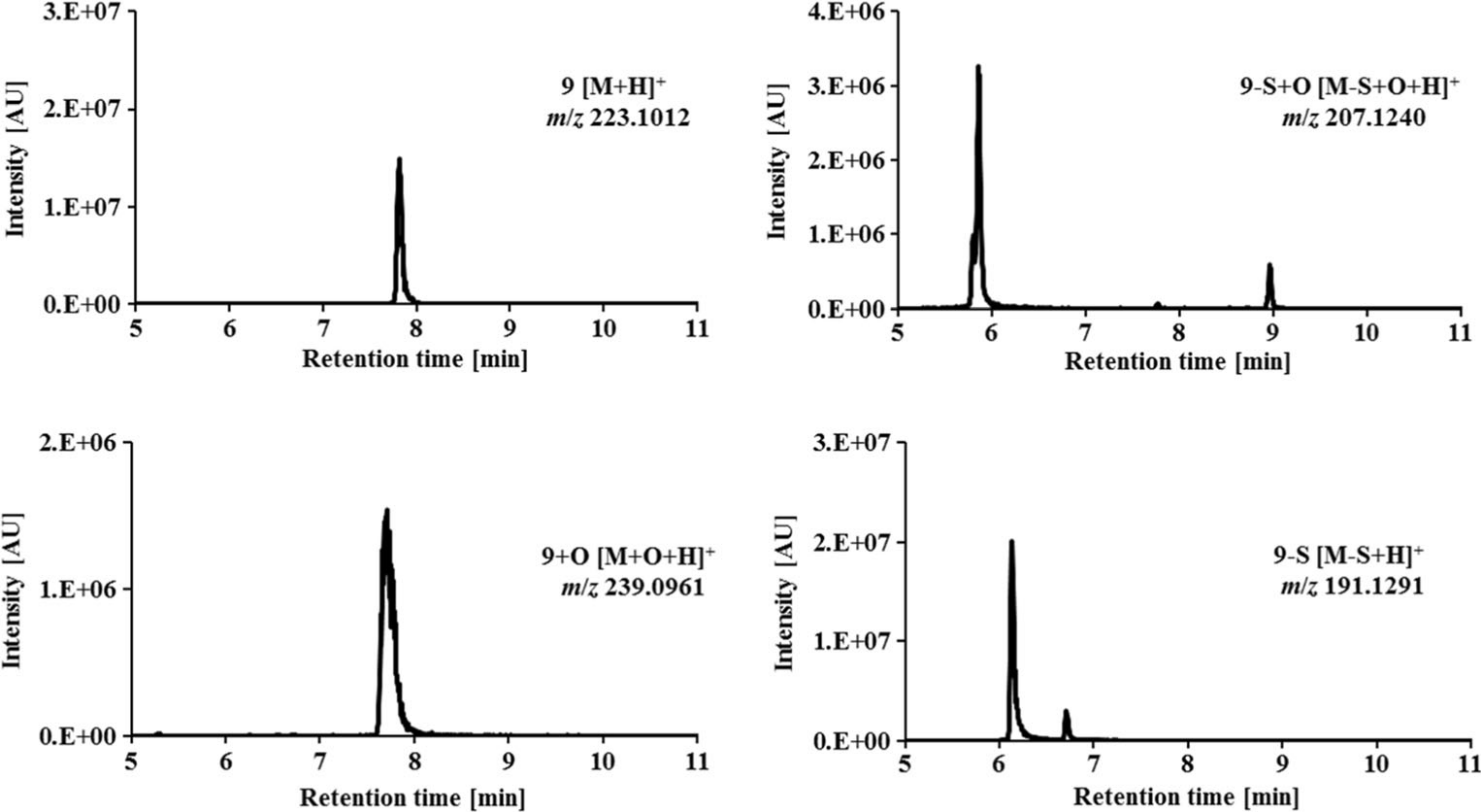
LC-HRMS analysis of 2-acetylpyridine *N*,*N*-dimethyl thiosemicarbazone (**9**) and its main metabolites after electrochemical oxidation at + 1800 mV vs. Pd/H_2_

As an example of the chromatographic separation of the oxidation products obtained from the mass voltammograms, the LC-HRMS analysis of the terminally monomethylated Triapine derivative (**1**; [M + H]^+^ *m*/*z* 210.0808) at *t*_r_ = 6.2 min and its main metabolite (the dehydrogenated [M–2H + H]^+^ *m*/*z* 208.0651) with a retention time at *t*_r_ = 8.2 min is shown in Fig. 3. In comparison, in Fig. 4 the separation of the 2-acetylpyridine *N*,*N*-dimethyl thiosemicarbazone **9** ([M + H]^+^ *m*/*z* 223.1012) together with its main metabolic products is depicted. One hydroxylated metabolite ([M + O + H]^+^ *m*/*z* 239.0961) was detected at *t*_r_ = 8.2 min. In case of oxidative desulfuration for both, the semicarbazone ([M-S + O + H]^+^ *m*/*z* 207.1240) and amidrazone ([M-S + H]^+^ *m*/*z* 191.1291), two isomers were obtained (semicarbazone: *t*_r_ = 5.9 min, *t*_r_ = 8.9 min and amidrazone: *t*_r_ = 6.2 min, *t*_r_ = 6.7 min). All other metabolites of **1** and **9** previously obtained by EC/MS were not detected by LC/MS. The list of all metabolic reactions and products of the investigated thiosemicarbazone derivatives **1**–**10** elucidated by means of EC/LC-ESI-HRMS is presented in Table 3, whereas the demethylated products are presented in Table 4.

**Table 3:** Metabolic reactions observed for the investigated thiosemicarbazones via LC-HRMS after electrochemical oxidation at + 1800 mV vs. Pd/H_2_ with the intensities of the obtained metabolites (**✓**: > 10^6^ AU, **~**: 10^5^–10^6^ AU, **x**: < 10^4^ AU)

Modification	Metabolic reaction	**1**	**2**	**3**	**4**	**5**	**6**	**7**	**8**	**9**	**10**
[M–2H + H]^+^	Dehydrogenation	**✓**	**✓**	**✓**	**✓**	**✓**	**✓**	**x**	**✓**	**x**	**✓**
[M + O + H]^+^	Hydroxylation	**~**	**✓**	**✓**	**✓**	**✓**	**✓**	**✓**	**~**	**✓**	**~**
[M + O–2H + H]^+^	Hydroxylation/Dehydrogenation	**x**	**✓**	**~**	**x**	**✓**	**x**	**x**	**✓**	**~**	**✓**
[M–S + H]^+^	Desulfuration C (Amidrazone)	**x**	**✓**	**✓**	**✓**	**✓**	**✓**	**✓**	**✓**	**✓**	**~**
[M–S–2H + H]^+^	Desulfuration/Dehydrogenation	**~**	**✓**	**~**	**✓**	**~**	**x**	**✓**	**~**	**~**	**✓**
[M–S + O + H]^+^	Desulfuration C=O (Semicarbazone)	**~**	**✓**	**x**	**~**	**~**	**~**	**x**	**✓**	**✓**	**~**
[M–S + O–2H + H]^+^	Desulfuration/Dehydrogenation	**~**	**✓**	**x**	**~**	**✓**	**✓**	**x**	**✓**	**x**	**✓**

**Table 4:**
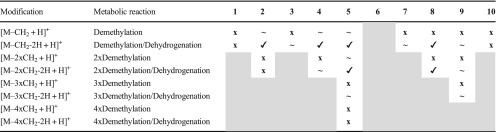
Demethylation of the investigated thiosemicarbazones detected via LC-HRMS after electrochemical oxidation at + 1800 mV vs. Pd/H_2_ with the intensities of the obtained metabolites (**✓**: > 10^6^ AU, **~**: 10^5^–10^6^ AU, **x**: < 10^4^ AU; the gray areas represent metabolic reactions which are not possible)

Overall, the most intensive metabolites from the mass voltammograms were also detected by LC-HRMS analysis. For all other metabolites, which were obtained by EC/MS but not via LC/MS, it can be assumed that they (1) were not present in sufficient concentrations for LC-MS detection or (2) their chemical stability was too low for separation (the time span between the electrochemical generation and detection is shorter in EC/MS compared to LC/MS and consequently also unstable, short-lived species can be observed in mass voltammograms). Hydroxylation was the only reaction which was observed with LC/MS for all the investigated compounds (in case of **1**, **8** and **10** only in low amounts). The dehydrogenated metabolites were detected for all thiosemicarbazones except the 2-acetylpyridine-containing **7** and **9**. Oxidative desulfuration to amidrazone was observed for all derivatives except compound **1**, whereas the respective semicarbazone was not observed in case of **3** and **7**. Demethylated metabolites were only observed for **2**, **4** and **5** in low levels, although all other compounds (except **6**) also possess methyl groups. Notably, many of these demethylated metabolites could not be detected because they rapidly undergo a further dehydrogenation step (this was observed for all compounds except **1** and **10**). In general, no metabolic trends could be observed for the direct Triapine-derivatives (compounds **1**–**5**, **10**) in comparison to compounds **6**–**9** without amino group at the pyridine ring.

### Microsomal incubations of thiosemicarbazones

**Fig. 5:**
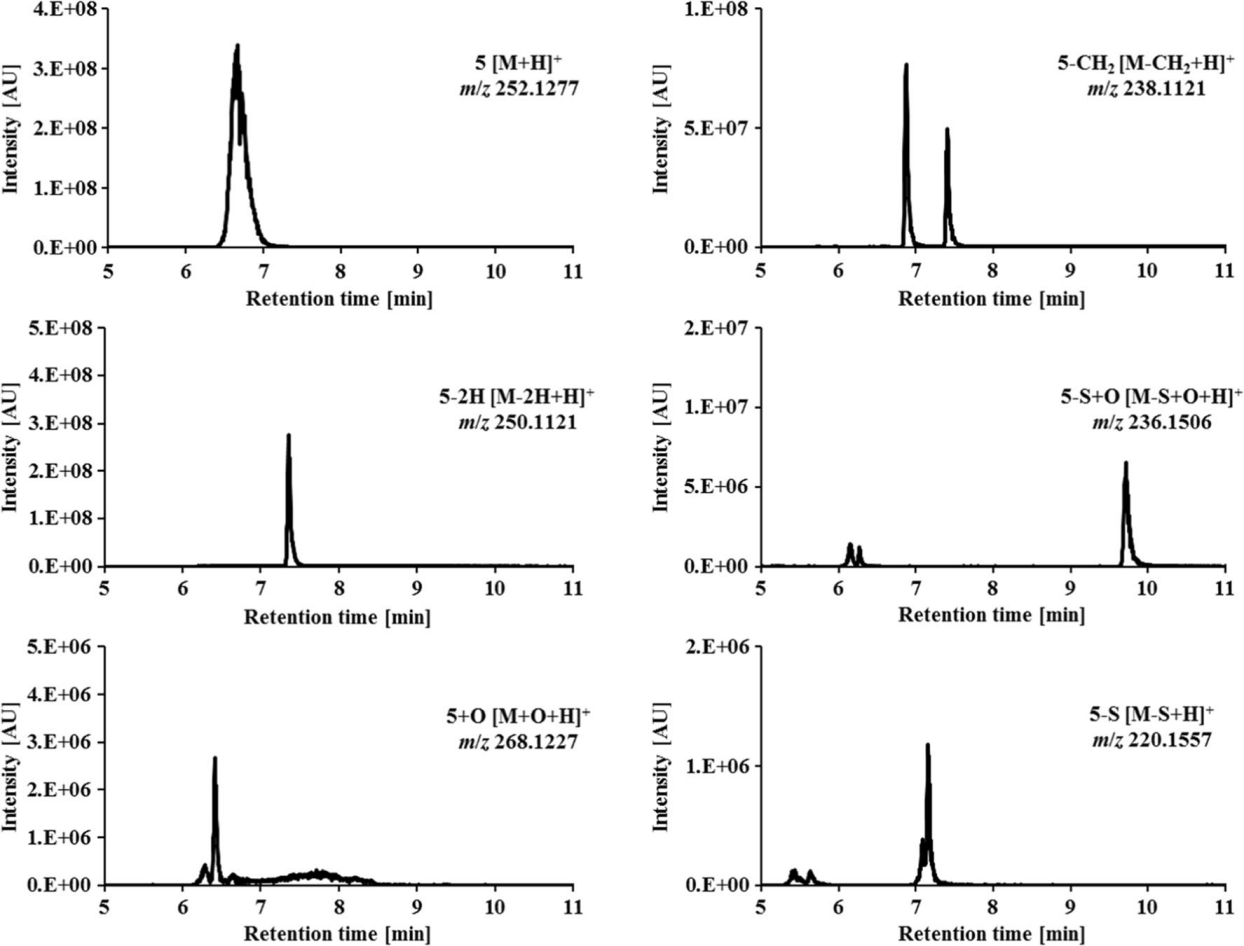
LC-HRMS analysis of the tetramethylated Triapine derivative **5** and some of its main metabolites after cell-free incubations with HLM

The second method for the analysis of the metabolic conversions of the thiosemicarbazones was cell-free incubation with human liver microsomes (HLM). To this end, the respective thiosemicarbazone was incubated with HLM for 2 h. Then, the metabolic reactions were stopped by addition of acetonitrile. Finally, the proteins were removed via centrifugation and the samples were analyzed by means of LC/ESI-HRMS. As an example of the performed metabolic conversions via microsomes, the chromatograms obtained for the tetramethylated Triapine derivative **5** and its main metabolic reactions are given in Fig. 5. Intact derivative **5** ([M + H]^+^ *m/z* 252.1277) eluted at *t*_r_ = 6.7 min, its dehydrogenated metabolite ([M–2H + H]^+^ *m/z* 250.1121) at *t*_r_ = 7.4 min and its hydroxylated metabolite ([M + O + H]^+^ *m*/*z* 268.1227) at *t*_r_ = 6.4 min, whereas two mono-demethylated isomers ([M–CH_2_ + H]^+^ *m/z* 238.1121) were separated at *t*_r_ = 6.9 min and *t*_r_ = 7.4 min. The respective isomers formed upon oxidative desulfuration were detected at *t*_r_ = 6.2 min and *t*_r_ = 9.7 min (for the semicarbazone [M–S + O + H]^+^ *m/z* 236.1506) and at *t*_r_ = 5.5 min and *t*_r_ = 7.1 min (for the amidrazone [M–S + H]^+^ *m/z* 220.1557), respectively. Notably, subsequent dehydrogenations of these metabolites, as well as a variety of additional two- and threefold demethylated metabolites, were observed as well (see Tables 5 and 6). All metabolic reactions of the investigated thiosemicarbazones, together with the intensities of the obtained products, are summarized in Table 5 (dehydrogenation, hydroxylation, oxidative desulfuration) and Table 6 (demethylation).

**Table 5:** Metabolic reactions observed for the investigated thiosemicarbazones via LC-HRMS after cell-free incubations with HLM with the intensities of the obtained metabolites (**✓**: > 10^6^ AU, **~**: 10^5^–10^6^ AU, **x**: < 10^4^ AU)

Modification	Metabolic reaction	**1**	**2**	**3**	**4**	**5**	**6**	**7**	**8**	**9**	**10**
[M–2H + H]^+^	Dehydrogenation	**✓**	**✓**	**✓**	**✓**	**✓**	**✓**	**x**	**✓**	**x**	**✓**
[M + O + H]^+^	Hydroxylation	**✓**	**✓**	**✓**	**✓**	**✓**	**✓**	**✓**	**✓**	**✓**	**✓**
[M + O–2H + H]^+^	Hydroxylation/Dehydrogenation	**✓**	**✓**	**✓**	**✓**	**✓**	**~**	**x**	**✓**	**x**	**✓**
[M–S + H]^+^	Desulfuration C (Amidrazone)	**✓**	**~**	**✓**	**✓**	**✓**	**✓**	**✓**	**✓**	**✓**	**✓**
[M–S–2H + H]^+^	Desulfuration/Dehydrogenation	**✓**	**x**	**✓**	**✓**	**~**	**✓**	**✓**	**x**	**~**	**✓**
[M–S + O + H]^+^	Desulfuration C=O (Semicarbazone)	**✓**	**~**	**~**	**✓**	**✓**	**✓**	**✓**	**~**	**✓**	**✓**
[M–S + O–2H + H]^+^	Desulfuration/Dehydrogenation	**✓**	**~**	**~**	**~**	**✓**	**~**	**x**	**~**	**x**	**✓**

**Table 6:**
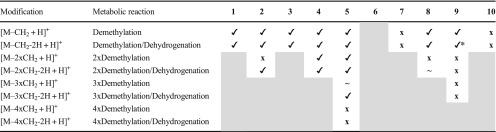
Demethylation of the investigated thiosemicarbazones detected via LC-HRMS after cell-free incubations with HLM with the intensities of the obtained metabolites (**✓**: > 10^6^ AU, **~**: 10^5^–10^6^ AU, **x**: < 10^4^ AU; the gray areas represent metabolic reactions which are not possible)

*The demethylated/dehydrogenated metabolite is not a product of the simply demethylated metabolite

Overall, the elucidation of metabolic pathways for the ten thiosemicarbazones upon cell-free incubations with HLM revealed strong differences among the investigated compounds. Thus, hydroxylated metabolites were detected for all derivatives. Dehydrogenation was observed in case of eight compounds (excluding **7** and **9)**, as well as for their respective semicarbazones and hydroxylated metabolic products. After oxidative desulfuration, for all ten compounds the subsequent amidrazone formation was observed (only in case of **2** in lower amounts), as well as the formation of the respective semicarbazone (lower amounts for **2**, **3** and **8**). Single demethylation was found for all compounds except **7** (with only a C-CH_3_ group), **6** (which does not bear a methyl group) and **10**. In comparison, a twofold demethylation was observed only for **4** and **5**, but for **2** and **8** the subsequent dehydrogenation occurred. Despite the presence of three methyl groups, **9** was only subject of a single demethylation. Finally, for the direct *N*-methylated Triapine derivatives the complete demethylation (resulting in formation of Triapine) was observed for **1**–**4** but not for the tetramethylated **5** and the N-N-CH_3_-bearing **10**.

### Structural elucidation of metabolites

**Fig. 6:**
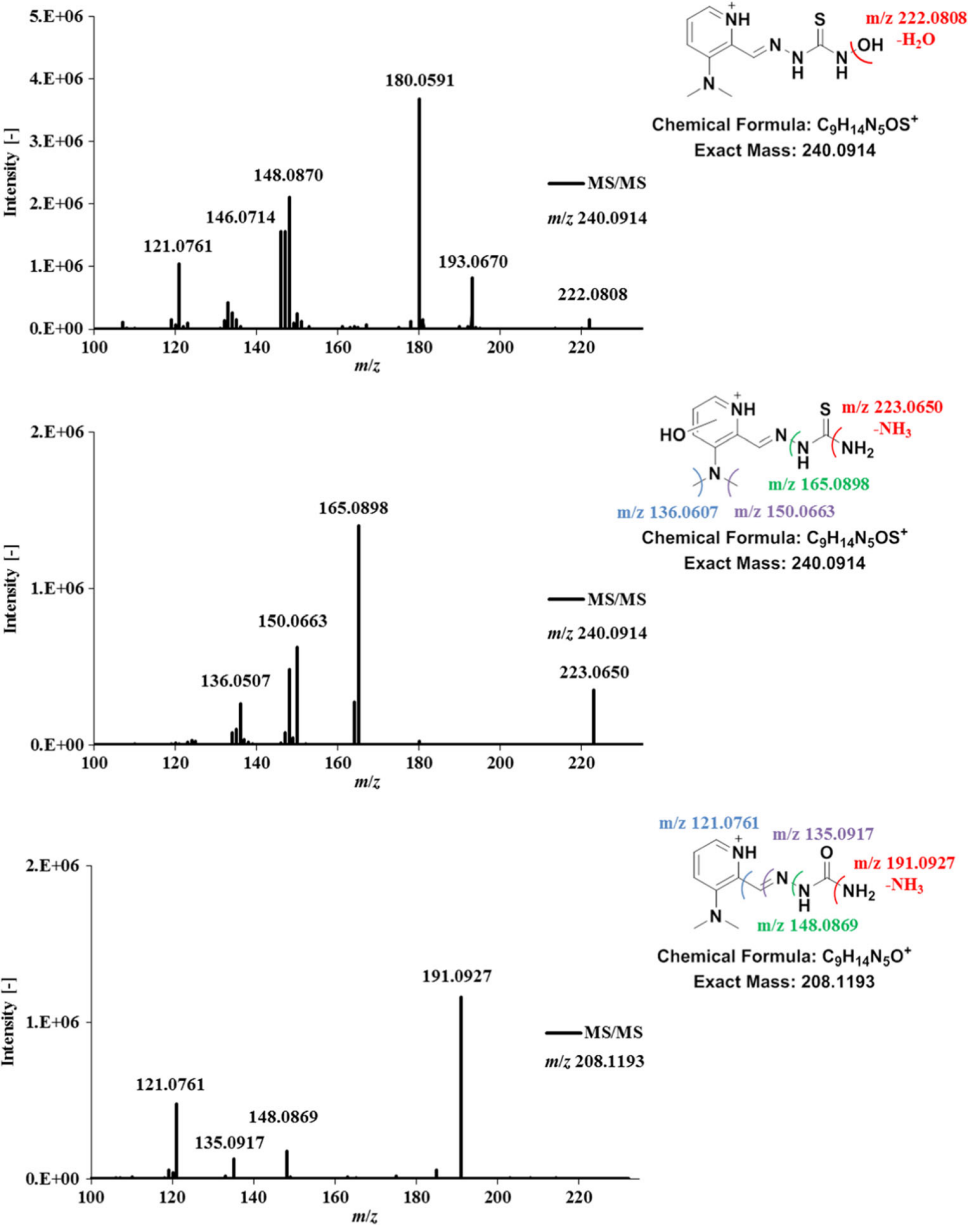
LC-MS/MS spectra and structural elucidation of the hydroxylation as well as semicarbazone formation of the 3-dimethylaminopyridine Triapine derivative **4** after incubation with human liver microsomes

**Fig. 7:**
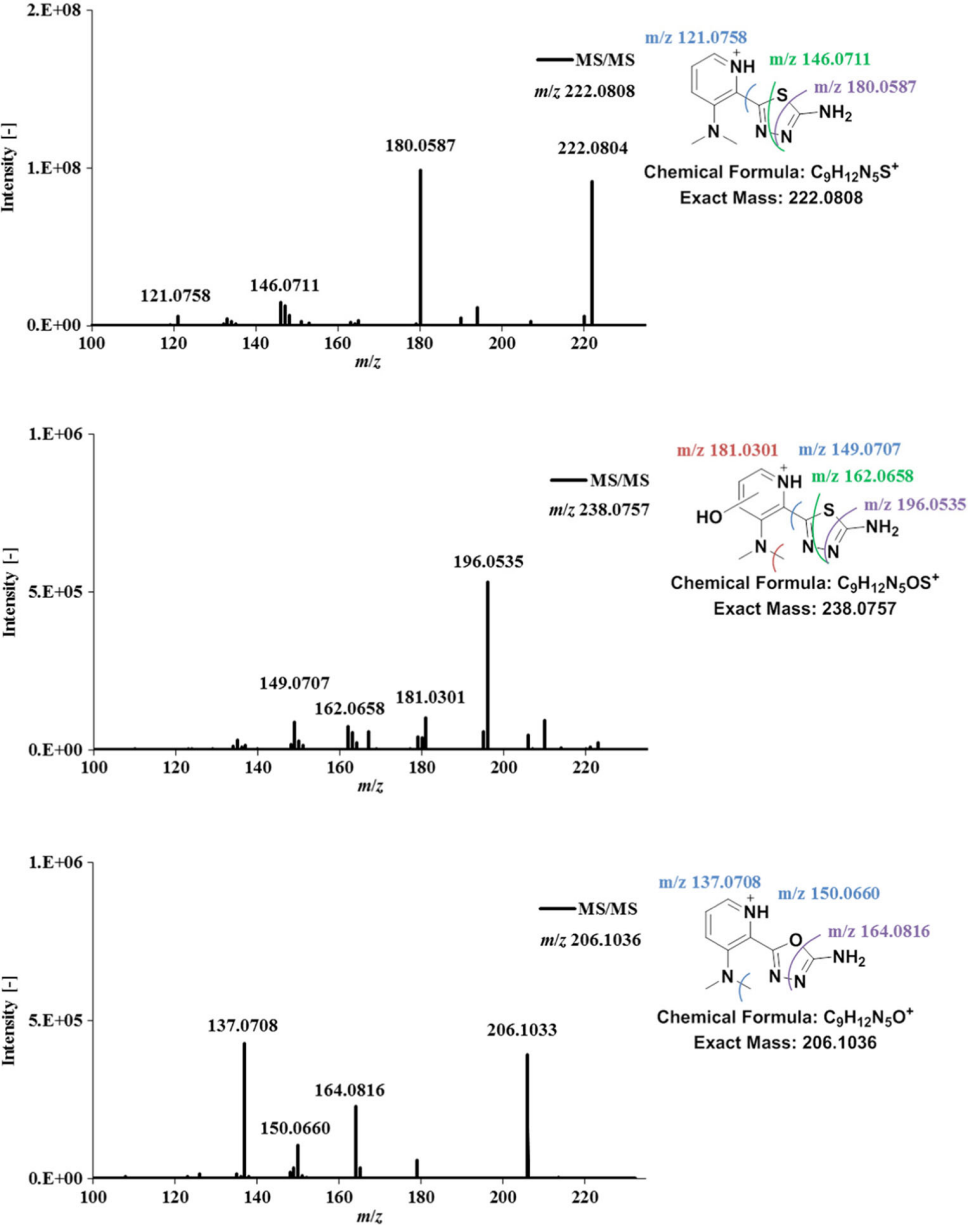
LC-MS/MS spectra and structural elucidation of the dehydrogenated metabolite of the 3-dimethylaminopyridine Triapine derivative **4** after incubation with human liver microsomes. In addition, also the dehydrogenated semicarbazone and dehydrogenated hydroxylated metabolites are depicted

For structural elucidation of the metabolites generated by microsomal incubation, MS/MS experiments were performed. As an example of the fragmentations and the obtained structures, the MS/MS spectra of the 3-dimethylaminopyridine Triapine derivative **4**, together with the assigned metabolites, are presented in Fig. 6. The fragmentation of intact **4** [C_9_H_14_N_5_S]^+^ *m/z* 224.0970 involved the formation of the highly intensive thiocarbonyl fragment [C_9_H_11_N_4_S]^+^ *m/z* 206.0699 upon loss of the terminal –NH_2_ moiety (data not shown). This is of interest, since in our previous work with Triapine the terminal deamination was crucial for the assignment of the different hydroxylated metabolites [18]. In detail, in case of a hydroxylation at the pyridine ring, the corresponding terminal deamination was observed. On the contrary, when the terminal –NH_2_ was hydroxylated with formation of the –NHOH hydroxylamine, the fragmentation pattern did not include the loss of –NH_3_ (Δ *m*/*z* 17) or –NH_2_OH (Δ *m*/*z* 33), which would again result in the thiocarbonyl [RC=S]^+^ fragment. Instead, the fragments showed loss of –H_2_O (Δ *m*/*z* 18) and no thiocarbonyl formation. Exactly the same pattern was also observed in case of hydroxylation of **4** ([M + O + H]^+^ *m*/*z* 240.0914). The isomer [C_9_H_14_N_5_SO]^+^ *m*/*z* 240.0914 at 6.2 min resulted in the fragment [C_9_H_12_N_5_S]^+^ *m*/*z* 222.0808 after loss of –H_2_O (Δ *m*/*z* 18) and, consequently, could be assigned to the hydroxylated terminal NH_2_–group (Fig. 6). Additionally, the detection of the MS/MS fragments [C_8_H_10_N_3_S]^+^ *m*/*z* 180.0587, [C_8_H_8_N_3_]^+^ *m*/*z* 146.0714 and [C_7_H_9_N_2_]^+^ *m*/*z* 121.0761 further supported the hypothesis of substitution at the terminal nitrogen and could be clearly assigned to the formation of the dehydrogenated ring-closed thiadiazole in the interface (see discussion below and Fig. 7). On the other hand, the MS/MS measurements of the hydroxylated species [C_9_H_14_N_5_SO]^+^ *m*/*z* 240.0914 at 6.5 min revealed the formation of the positively charged thiocarbonyl fragment [C_9_H_11_N_4_SO]^+^ *m*/*z* 223.0650 after terminal deamination and loss of –NH_3_ (Δ *m*/*z* 17), which supports its attribution to the isomer with the –OH directly at the pyridine ring. This structure could also be confirmed by additional fragments [C_8_H_11_N_3_O]^+^ *m*/*z* 165.0898, [C_7_H_8_N_3_O]^+^ *m*/*z* 150.0663 and [C_6_H_6_N_3_O]^+^ *m*/*z* 136.0507, detectable only in case of a pyridine hydroxylation (Fig. 6). The just mentioned fragmentation pattern was relevant also in case of the oxidative desulfuration to the semicarbazone ([M–S + O + H]^+^ *m*/*z* 208.1193), which was confirmed by the fragments [C_9_H_11_N_4_O]^+^ *m*/*z* 191.0927 upon terminal deamination and loss of –NH_3_ (Δ *m*/*z* 17), [C_8_H_10_N_3_]^+^ *m*/*z* 148.0869 (loss of urea), [C_8_H_11_N_2_]^+^ *m*/*z* 135.0917 (loss of semicarbazide) as well as [C_7_H_9_N_2_]^+^ *m*/*z* 121.0761 (Fig. 6).

In case of dehydrogenation [M–2H + H]^+^, the only plausible structures involve ring formation, either to a 1,2,4-triazole or 1,3,4-thiadiazole. In our previous work, the MS/MS spectra of dehydrogenated Triapine suggested generation of the thiadiazole (compare Scheme 1) which was finally confirmed also via NMR. The fragment [C_6_H_6_N_3_S]^+^ with loss of N=C–NH_2_ was crucial for this identification, since it can be formed only in case of a thiadiazole. This fragmentation was also observed in case of the dehydrogenated **4** [C_9_H_12_N_5_S]^+^ *m*/*z* 222.0808 with generation of [C_8_H_10_N_3_S]^+^ *m*/*z* 180.0587 which also proved the thiadiazole structure formation (Fig. 7, additional fragments [C_8_H_8_N_3_]^+^ *m*/*z* 146.0711 and [C_7_H_9_N_2_]^+^ *m*/*z* 121.0758). Analogously, also for the pyridine ring-hydroxylated dehydrogenated species [M + O–2H + H]^+^ ([C_9_H_12_N_5_OS]^+^ *m*/*z* 238.0757) the thiadiazole formation could be confirmed with the fragment [C_8_H_10_N_3_SO]^+^ *m*/*z* 196.0535 (Fig. 7). The same was observed for the dehydrogenated semicarbazone [M–S + O–2H + H]^+^ ([C_9_H_12_N_5_O]^+^ *m*/*z* 206.1036) with occurrence of [C_8_H_10_N_3_O]^+^ *m*/*z* 164.0816 proving the formation of an oxadiazole (Fig. 7, additional fragments [C_7_H_8_N_3_O]^+^ *m*/*z* 150.0660 and [C_7_H_9_N_2_O]^+^ *m*/*z* 137.0708). In case of the dehydrogenated amidrazone [M–S–2H + H]^+^ ([C_9_H_12_N_5_]^+^ *m/z* 190.1087) with a suggested ring-closed triazole structure, the fragmentation pattern was very similar to the amidrazone [M–S + H]^+^ ([C_9_H_14_N_5_]^+^ *m/z* 192.1243) itself. Consequently, the MS/MS spectra could not prove the proposed structure (data not shown).

**Scheme 3:**
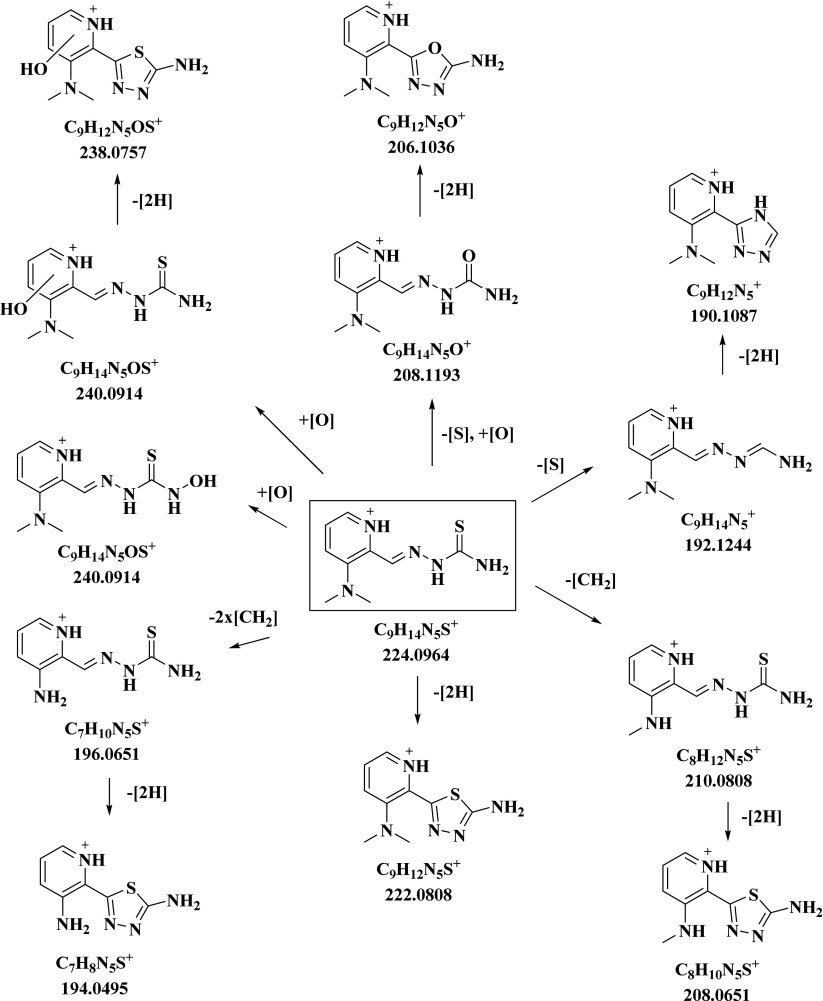
Overview of the metabolic pathways of the 3-dimethylaminopyridine Triapine derivative **4** elucidated by LC-HRMS after incubation with cell-free human liver microsomes

Furthermore, during the microsomal incubation compound **4** underwent additionally single demethylation [M–CH_2_ + H]^+^ and twofold demethylation [M–2xCH_2_ + H]^+^, as well as the subsequent dehydrogenation reactions to [M–CH_2_–2H + H]^+^ and [M–2xCH_2_–2H + H]^+^. Notably, single demethylation of **4** resulted in the formation of **3** [C_8_H_12_N_5_S]^+^ *m*/*z* 210.0808. This metabolic reaction was confirmed by the same retention time of these two molecules as well as by their identical MS/MS spectra. Accordingly, the demethylated/dehydrogenated metabolite of **4** [C_8_H_10_N_5_S]^+^ *m*/*z* 208.0651 was identical to the dehydrogenated thiadiazole metabolite of **2**. Furthermore, after a twofold demethylation of **4,** the generation of Triapine [C_7_H_10_N_5_S]^+^ *m*/*z* 196.0651 and its dehydrogenated ring-closed thiadiazole metabolite [C_7_H_8_N_5_S]^+^ *m*/*z* 194.0495 was observed (Scheme 1). An overview of the metabolic pathways of **4** is given in Scheme 3, whereas the metabolites of the other nine thiosemicarbazones **1**–**3** and **5**–**10** are presented in Schemes S2-S10 in the ESM.

### Metabolic reactions of **5** in vivo

**Scheme 4:**
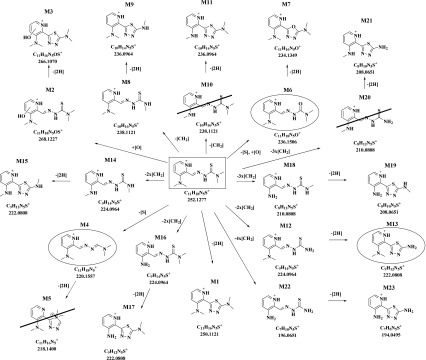
Overview of the in vivo metabolic pathways of **5** (main metabolites are marked with a circle; metabolites observed with microsomes but not in vivo are crossed out)

**Fig. 8:**
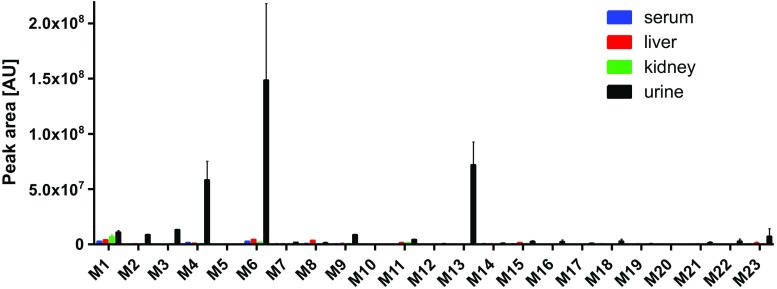
Relative quantification of metabolites 1–23 of compound **5** in serum/liver/kidney/urine samples of mice

Our recent metabolic data of Triapine in vivo showed very rapid metabolisation and excretion of both intact Triapine and its metabolites [18]. In this study, the aim was to investigate in vivo one Triapine derivative with a different metabolic profile. However, from the aminopyridine compounds only **5** and **10** showed no generation of Triapine or any of its metabolites in the microsomal incubation studies. As compound **10** is biologically completely inactive (data not shown) and **5** is highly cytotoxic on cancer cell lines in the nanomolar range, we selected **5** for the in vivo investigations. Samples (serum, liver, kidney, urine) were collected 15 min after *i.v.* mice treatment with 5 mg/kg of **5** and acetonitrile was used for the extraction of the analytes, followed by their analysis via LC/ESI-HRMS. Most of the 21 metabolites which were observed by the microsomal incubations could also be found in the in vivo samples. Only the triazole formed after dehydrogenation of the amidrazone (M5) and two demethylated species (M10 and M20) were not observed (however, the respective dehydrogenated products M11 and M21). Additionally, also amounts of Triapine (M22) and its dehydrogenated thiadiazole (M23) were detected. Overall, the most abundant metabolites were the amidrazone M4, the semicarbazone M6, and the dimethylated/dehydrogenated M13. By far the highest amounts of the metabolites were observed in the urine. Additionally, the collected in vivo samples were screened for the phase II transformations glucuronidation, methylation, sulfation and acetylation as well as glutathione/glycine conjugation. However, only in urine traces of *N*-glucuronides and *O*-glucuronides were found (data not shown). An overview of the metabolic pathways and relative quantities of the metabolites is depicted in Scheme 4 and Fig. 8 (a zoom of Fig. 8 with more details of the minor species can be found in ESM Fig. S9).

## Discussion

5-HP and Triapine are so far the only two α-*N*-heterocyclic thiosemicarbazones which completed some clinical investigations [13, 19, 20]. However, 5-HP was not further clinically developed due to the rapid inactivation via glucuronidation [19, 20]. Also for Triapine, which was extensively studied in clinical trials, a short plasma half-life time was reported together with a very rapid metabolisation and excretion in our recent studies in mice [17, 18]. Hence, both compounds are featured by unfavorable ADME properties, which seem to be at least partially responsible for their inefficiency. In order to understand the structure/metabolism relationships of α-*N*-heterocyclic thiosemicarbazones in more detail, in this study ten derivatives of Triapine were investigated and their metabolic profiles were elucidated by application of (i) electrochemical oxidation and (ii) microsomal incubations, followed by their analysis via (LC-)HRMS.

In general, high correlations of the metabolic pathways were observed between electrochemical oxidation, as the purely instrumental technique, in comparison to cell-free microsomal incubations. This was especially remarkable for the main metabolic reactions dehydrogenation, hydroxylation and oxidative desulfuration to semicarbazone/amidrazone. The subsequent dehydrogenation of these metabolites was observed in lower extent by EC/MS compared to microsomes. Demethylation (and its subsequent dehydrogenation) was the only reaction which was generated mainly by the application of microsomes and was hardly observed with EC/MS. To facilitate the comparison of the observed similarities and differences among the investigated panel of thiosemicarbazones, in the following paragraphs, only the metabolic conversions obtained via cell-free incubations with microsomes will be discussed.

i) **Dehydrogenation**
  The only chemically meaningful structures for the dehydrogenation of the investigated thiosemicarbazones is a ring-closing reaction leading to the formation of either a 1,3,4-thiadiazole or a 1,2,4-triazole. In our previous work, we could confirm the thiadiazole structure for Triapine by fragmentation experiments as well as by NMR spectra of the chemically synthesized dehydrogenated metabolite [18]. Also in the here presented study dehydrogenation was observed (for all except **7** and **9**) and the formation of the thiadiazole could be confirmed by detected fragments which can be generated only in case of this ring-closed structure (loss of N=C–NR_2_, see Fig. 7 for **4** [C_8_H_10_N_3_S]^+^ *m*/*z* 180.0587). Notably, for the dehydrogenated **10** ([C_8_H_10_N_5_S]^+^ *m*/*z* 208.0651), a thiadiazole ring with a positively charged nitrogen was suggested due to the presence of the methyl group at the hydrazinic NH–moiety (ESM Scheme S10). In case of **7** and **9**, thiadiazole formation could not be observed neither directly nor with a positively charged nitrogen. This can be explained as both compounds bear a methyl group instead of a hydrogen at the C=N bond, which disables the nucleophilic attack of the sulfur. Triazole formation was not observed in any of the derivatives, also not in case of **7** where thiadiazole formation is not possible (terminal dimethylation of **9** prevents ring-formation in general). This suggests that the formation of thiadiazoles in general is highly favored. A likely explanation is the much stronger nucleophilic character of the sulfur compared to the amide-like terminal thiosemicarbazide nitrogen, which is deactivated by the electron-withdrawing C=S thiocarbonyl.
ii) **Hydroxylation**
  Among the investigated panel, hydroxylation was the only metabolic reaction which was detected in high amounts for all compounds. Although there are different possibilities for oxygen insertion (*N*-oxidation or *C*-/*N*-hydroxylation), only hydroxylations of the terminal –NH_2_ group or *C*-hydroxylations directly at the pyridine ring seem to be formed. As already mentioned in the previous sections, the molecular ion [M + H]^+^ of thiosemicarbazones is always accompanied by its positively charged thiocarbonyl fragment [RC=S]^+^ upon dissociation of terminal –NH_2_/–NHR/–NR_2_ (only in case of **10** with a methylated hydrazinic NH next to the –C(=S) moiety this was not observed). This thiocarbonyl fragment was crucial for the assignment of the detected different hydroxylated isomers. Notably, only in case of hydroxylation of the terminal –NH_2_ group with formation of the –NHOH hydroxylamine, this thiocarbonyl pattern was not observed. Moreover, upon fragmentation of these terminally hydroxylated –NHOH metabolites, loss of Δ *m*/*z* 18 (-H_2_O) was observed with a simultaneous ring-formation of the respective thiadiazole in the interface (confirmed by the same fragmentation pattern as in case of a dehydrogenation to the thiadiazole). Notably, this terminal hydroxylamine formation was observed for all thiosemicarbazones except **2**, **5**, **8** and **9** with a terminally dimethylated –NR_2_ moiety, which strongly supports this assignment. On the other hand, if the hydroxylation takes place directly at the pyridine ring, the corresponding [RC=S]^+^ fragment was always detectable. Regarding the exact chemical structures, the hydroxylation can take place at all three (compound **1**–**5** and **10**) or four (compound **6**–**9**) carbon atoms of the pyridine ring. Unfortunately, based on their high chemical similarity, their retention times do not always sufficiently differ for separation (see hydroxylated metabolites of **5** in Fig. 5) or, probably even co-elute (as an example see hydroxylation of **9** in Fig. 4). Additionally, their MS/MS spectra are identical. Thus, the determination of the total number of *C*(pyridine)-hydroxylated metabolites of the investigated thiosemicarbazone as well as of the exact OH-position at the ring was not possible.
  Upon *C*-hydroxylation at the pyridine ring, also a subsequent dehydrogenation was observed for all investigated compounds (also here with the exception of **7** and **9** as discussed above). Again, the ring-closure reaction with formation of the thiadiazole could be confirmed by the MS/MS fragments with loss of N=C–NR_2_ for all these metabolites. As expected, for the structural isomers with different OH-positions at the ring the respective ring-closed dehydrogenated metabolites all showed the same MS/MS pattern. Notably, in case of the *N*-hydroxylated metabolites no dehydrogenation was observed.
iii) **Oxidative desulfuration**
  Oxidative desulfuration is a metabolic reaction in which a thiocarbonyl group of a thiosemicarbazone R–NH–C(=S)–NR_2_ is oxidized to a carbonyl group R–NH–(C=O)–NR_2_ with formation of a semicarbazone, or via R–NH–C(=SO_2_)–NR_2_ generates the R–N=CH–NR_2_ amidrazone upon SO_2_–dissociation [33, 34]. In general, oxidative desulfuration was observed for all investigated compounds, although the semicarbazone of **2**, **3** and **8**, as well as the amidrazone of **2**, were generated only in low amounts.
  With regard to the general metabolite [M-S + O], also other structural isomers are conceivable which do not involve a direct substitution of C=S to C=O (as in case of semicarbazone). Also a combination of metabolic reactions, such as hydroxylation of an amidrazone, would result in the same exact mass as the semicarbazone. However, all observed [M–S + O] metabolites showed MS/MS spectra which involve the formation of the positively charged carbonyl cation [C=O]^+^ and further fragments with loss of NH–C(=O)–NR_2_, as well as N–NH–C(=O)–NR_2_, proved the semicarbazone structure (in Fig. 6 all these fragments are depicted for the semicarbazone of **4** [C_9_H_14_N_5_O]^+^ *m*/*z* 208.1193). These semicarbazones (as well as the amidrazones [M–S]) additionally formed isomers (see Figs. 4 and 5) which can most likely be explained by the respective *E*/*Z*-isomerism, a phenomenon which is well-known for the substance class of thiosemicarbazones [27]. In case of dehydrogenation after oxidative desulfuration, the crucial fragment with loss of N=C–NR_2_ was observed once again, thereby confirming the formation of a ring-closed oxadiazole. This again, as in case of sulfur, shows that the ring-closure is initiated by the higher nucleophilic carbonyl oxygen compared to the electron-deficient terminal nitrogen (which would result in triazole formation). Only in case of oxidative desulfuration to the amidrazone, the following dehydrogenation resulted in a triazole structure, which can be explained by the higher nucleophilic character of the terminal nitrogen in the absence of sulfur or oxygen (thioamide/amide → amine), enabling the induction of a ring-closure. This triazole was observed for all compounds except for the terminally dimethylated compounds **2** and **8**. Although unexpected, for the two other terminally dimethylated compounds **5** and **9**, such a ring-closure was observed. In case of **5** this reaction can be explained by formation of positively charged quarternary nitrogen (ESM Scheme S5). In case of 2-acetylpyridine *N,N*-dimethyl thiosemicarbazone (**9**), the observed exact mass of a dehydrogenated amidrazone *m/z* 189.1140 could not be assigned, despite MS/MS experiments, to a meaningful structure.
iv) **Demethylation**
  The demethylation pattern of the different thiosemicarbazones was of high interest, considering that several of the investigated substances upon demethylation can be converted to other substances of the panel. Furthermore, demethylation is the only reaction which cannot occur for Triapine itself. In addition, six (**1**–**5** and **10**) of the studied derivatives can, after complete demethylation, generate Triapine (and after subsequent dehydrogenation also the thiadiazole of Triapine). In detail, the demethylation of the singly methylated Triapine **1** (terminal –NH–CH_3_) and **3** (pyridine –NH–CH_3_) generated Triapine, and by subsequent dehydrogenation also the thiadiazole of Triapine (M1: Triapine–2H, Scheme 1). Their corresponding dimethylated derivatives **2** (terminal –N–(CH_3_)_2_) and **4** (pyridine –N–(CH_3_)_2_) underwent single demethylation (to **1** and **3**) and dehydrogenation (to the thiadiazoles of **1** and **3**). A twofold demethylation to Triapine and additional dehydrogenation to the thiadiazole of Triapine was observed only for **4**. Single demethylation of the tetramethylated **5** was observed once for each amino group leading to formation of both, terminal –NH–CH_3_/pyridine –N–(CH_3_)_2_ and terminal –N–(CH_3_)_2_/pyridine –NH–CH_3_ (together with the corresponding thiadiazoles). Twofold demethylation of **5** formed **2**, **4** and a third dimethylated structure with terminal –NH–CH_3_/pyridine –NH–CH_3_ (again with their thiadiazoles). The threefold demethylation of **5** led to the formation of singly methylated **1** and **3** and to their thiadiazoles. The fourfold demethylation to Triapine or its thiadiazole was not detected. The formation of all these metabolites was confirmed by MS/MS spectra and the retention times of the LC-HRMS analysis of the metabolites were identical to the respective synthesized compounds. Notably, for **10** no demethylation or demethylation/dehydrogenation was observed, suggesting that the hydrazinic NH–CH_3_ is the only position in the Triapine structure which is resistant to demethylation. **6** is the only derivative among the investigated panel which does not bear any methyl group, but can be generated by complete demethylation of **7**–**9**. Surprisingly, only its dehydrogenated metabolite (thiadiazole) was generated by complete demethylation and dehydrogenation of **8** (ESM Scheme S8), again confirmed by the comparison of their retention times and MS/MS spectra (also the singly demethylated compound of **8** was observed). Interestingly, for **9**, despite three methyl groups, only single demethylation at the terminal –N(CH_3_)_2_ was detected (ESM Scheme S9). The other possible product after demethylation of the (CH_3_)–C=N was not formed. However, unexpectedly, its subsequently dehydrogenated metabolite was observed and could be proved by the same retention time and identical MS/MS spectra as the thiadiazole of **8**. In contrast, for **7** this C–CH_3_ demethylation or demethylation/dehydrogenation were not observed.

In comparison to the metabolic reactions observed for the thiosemicarbazones **1**–**10** investigated in this study, in case of Triapine the formation of the semicarbazone and amidrazone (metabolites upon oxidative desulfuration) were not observed via incubations with cell-free microsomes [18]. In literature, so far only cell-free microsomal incubation of the terminally *N*-dialkylated DpC and terminally mono *N*-ethylated Bp4eT (2-benzoylpyridine 4-ethyl-3-thiosemicarbazone; ESM Scheme S1) Richardson-type thiosemicarbazones were reported. Both resulted mainly in the formation of hydroxylated species and after oxidative desulfuration of the semicarbazone and amidrazone [24, 35, 36]. In case of DpC, also demethylation was observed, however, no deethylation for Bp4eT. Dehydrogenation was not observed for both Richardson-type compounds. This is in very good agreement with our thiosemicarbazones **7** and **9**, which are also substituted at the imine-carbon and in accordance with the assumption that hydrogen at this position is required for dehydrogenation and ring closure.

Regarding the biological activity of the investigated compounds, our panel contained thiosemicarbazones with (i) cytotoxic activities (**1**–**4**, **6** and **7**) comparable to Triapine (IC_50_ ~ 0.5 μM) [26, 27], (ii) strongly enhanced activity in the nanomolar range (**5**, **8** and **9**) [25–27] and (iii) the completely inactive compound **10** (unpublished data). In case of Triapine, our recent investigations showed that the dehydrogenation with simultaneous ring-closing reaction to the thiadiazole (also a main metabolite in vivo) resulted in complete biological inactivation (IC_50_ > 100 μM) [18]. The underlying explanation is most probably that this ring-closure destroys the *N*,*N*,*S-*donor set arrangement, which is essential for the biological mode of action involving iron binding prior to RR inhibition. In this study for **7** and **9**, possessing the CH_3_-C=N moiety, this metabolic reaction was not observed. Nevertheless, no significant difference in the cytotoxic activity to the direct H-C=N analogues **6** and **8**, which showed the thiadiazole formation, could be observed in cell culture [26]. Among the direct Triapine derivatives **1**–**5** and **10**, only **5** is cytotoxic in the nanomolar range. However, the metabolic profile did not significantly differ from the other derivatives. Consequently, it seems that the nanomolar activity of some derivatives cannot be correlated (at least with cell culture data) to its metabolic pathways. Notably, in case of all compounds where the *N*-methylation pattern results in in vitro cytotoxicities in the nanomolar range, their metabolic pathways showed demethylation process(es) which lead to the formation of derivatives with much lower cytotoxicity in the micromolar range [27]. Consequently, it was of high interest to study the metabolisation of one of these compounds (**5**) in vivo. In general, the metabolites obtained by electrochemical oxidation and cell-free microsomes could be confirmed by the in vivo samples. Only three of the metabolites were not observed: the triazole M5 and two demethylated species M10 and M20 (the respective dehydrogenated products M11 and M21 were detectable). Interestingly, additionally Triapine and its dehydrogenated thiadiazole were obtained resulting from complete demethylation of **5**, a transformation which was not observed via electrochemistry or microsomes. In comparison to Triapine, strong differences were observed: dehydrogenation and hydroxylation were the main metabolic reactions of Triapine in vivo, whereas **5** was converted predominantly to the amidrazone M4 and semicarbazone M6 as a result of oxidative desulfuration as well as the terminally double demethylated dehydrogenated M13 (Scheme 4). In serum, liver and kidney, the main metabolites of **5** were the dehydrogenated M1 and the semicarbazone M6 (in liver also terminally mono-demethylated M8). However, compared to the amounts of M4, M6 and M13 in urine the levels were very low (Fig. 8). Considering that the samples were taken 15 min after mice treatment, a very rapid metabolism and excretion can be concluded for **5**. In addition, all three main metabolites lost the crucial *N*,*N*,*S* functionality for metal ion chelation essential for the anticancer activity of α-*N*-heterocyclic thiosemicarbazones (semicarbazone with a *N*,*N*,*O*-donor set are usually much less active [37, 38]). Furthermore, as mentioned above any demethylation of the nanomolar cytotoxic **5** generates compounds with decreased cytotoxicities in the micromolar range [27]. Therefore, it can be assumed that the metabolisation of **5** results exclusively in compounds with much lower or no biological activity (in terms of IC_50_ values of cell culture experiments). Also for DpC and Bp4eT the main metabolites formed in vivo in rats were the respective products of oxidative desulfuration: amidrazone and semicarbazone [24, 36] (analogous metabolites to M13 cannot be formed). However, in case of the terminally dimethylated Dp44mT (ESM Scheme S1) as main metabolite the mono-demethylated compound was detected in vivo [24]. Consequently, although Dp44mT and **5** both possess a terminal *N*-dimethylation, the main metabolites are completely different, which suggests that already small modifications at the thiosemicarbazone structure can strongly impact the metabolisation process.

Taken together, the metabolic data of the here investigated panel of thiosemicarbazone clearly confirmed the assignments and structures of the metabolites of Triapine, especially regarding the ring-closing thiadiazole formation. Several distinct differences between the particular thiosemicarbazones could be observed, although a direct correlation between the anticancer activities and the metabolic profiles was not possible. Also in vivo different metabolic pathways were observed for the tetramethylated **5** compared to Triapine. However, the very fast metabolisation and excretion was similar. Further in vivo (therapy) experiments have to prove if e.g. the fast metabolisation of **5** indeed results in reduced anticancer activity and the exchange of methyl groups e.g. by piperidines or pyrrolidines (which cannot undergo simple metabolic demethylation) prevents the fast biological deactivation.

## Electronic supplementary material

ESM 1 (PDF 1530 kb)
