# Supplementary material for: Comparison of metabolic pathways of different α-N-heterocyclic thiosemicarbazones
Source: Anal Bioanal Chem. 2018 Feb 23;410(9):2343–61. doi: 10.1007/s00216-018-0889-x (PMC5849672; doi:10.1007/s00216-018-0889-x)
Supplement: Supplementary file 1 — (PDF 1530 kb) [file 216_2018_889_MOESM1_ESM.pdf]

**Analytical and Bioanalytical Chemistry**

**Electronic Supplementary Material**

# Comparison of metabolic pathways of different α-*N*-heterocyclic thiosemicarbazones

Karla Pelivan, Lisa M. Frensemeier, Uwe Karst, Gunda Koellensperger, Petra Heffeter, Bernhard K. Keppler, Christian R. Kowol

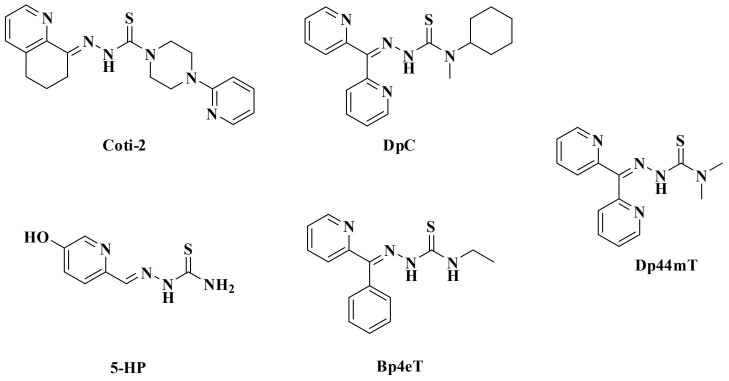


**Scheme S1** Molecular structures of Triapine, Coti-2, DpC, 5-HP, Dp44mT and Bp4eT

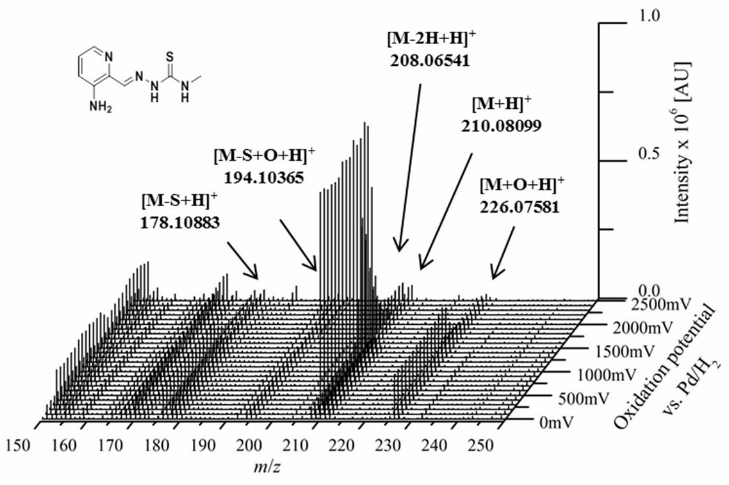


**Fig. S1** Mass voltammogram of thiosemicarbazone **1**

**Table S1** Metabolites of compound **1** detected by EC/MS

| Modification | Sum formula | Detected *m/z* | Theoretical *m/z* | Rel. deviation [ppm] | Metabolic reaction |
|---|---|---|---|---|---|
| $[M+H]^+$<br>$[M–NH–CH_3]^+$ | $C_8H_{12}N_5S$<br>$C_7H_7N_4S$ | 210.08099<br>179.03859 | 210.08079<br>179.03873 | 0.94<br>0.76 | – |
| $[M–2H+H]^+$ | $C_8H_{10}N_5S$ | 208.06541 | 208.06514 | 1.29 | Dehydrogenation |
| $[M–4H+H]^+$ | $C_8H_8N_5S$ | 206.04944 | 206.04949 | 0.26 | Dehydrogenation |
| $[M+O+H]^+$ | $C_8H_{12}N_5SO$ | 226.07581 | 226.07571 | 0.45 | Hydroxylation |
| $[M+O–2H+H]^+$ | $C_8H_{10}N_5SO$ | 224.06027 | 224.06006 | 0.95 | Hydroxylation/ Dehydrogenation |
| $[M–S+O+H]^+$ | $C_8H_{12}N_5O$ | 194.10365 | 194.10363 | 0.07 | Desulfuration |
| $[M–S+O–2H+H]^+$ | $C_8H_{10}N_5O$ | 192.08795 | 192.08799 | 0.19 | Desulfuration/ Dehydrogenation |
| $[M–S+H]^+$ | $C_8H_{12}N_5$ | 178.10883 | 178.10872 | 0.61 | Desulfuration |
| $[M–S–2H+H]^+$ | $C_8H_{10}N_5$ | 176.09323 | 176.09307 | 0.90 | Desulfuration/ Dehydrogenation |
| $[2M–2H+H]^+$ | $C_{16}H_{21}N_{10}S_2$ | 417.13864 | 417.13866 | 0.05 | Disulfide formation |
| $[2M–4H+H]^+$ | $C_{16}H_{19}N_{10}S_2$ | 415.12299 | 415.12301 | 0.05 | Disulfide formation/ Dehydrogenation |
| $[2M–6H+H]^+$ | $C_{16}H_{17}N_{10}S_2$ | 413.10745 | 413.10736 | 0.22 | Disulfide formation/ Dehydrogenation |

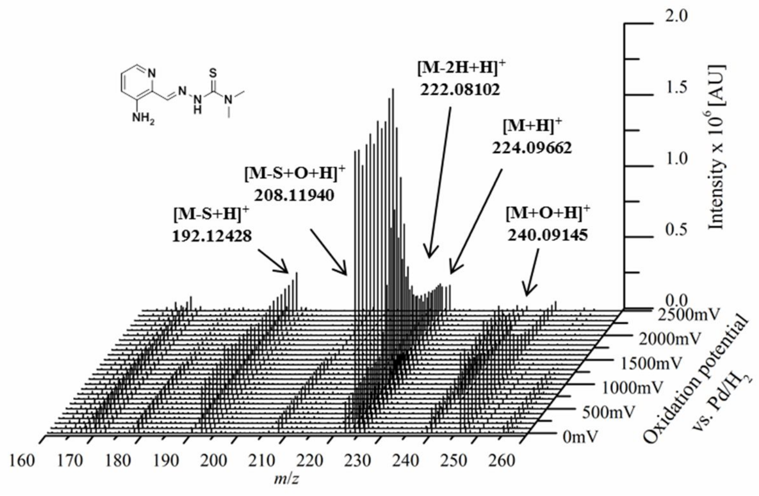


**Fig. S2** Mass voltammogram of thiosemicarbazone **2**

**Table S2** Metabolites of compound **2** detected by EC/MS

| Modification | Sum formula | Detected *m*/*z* | Theoretical *m*/*z* | Rel. deviation [ppm] | Metabolic reaction |
|---|---|---|---|---|---|
| $[M+H]^+$<br>$[M–N–2xCH_3]^+$ | $C_9H_{14}N_5S$<br>$C_7H_7N_4S$ | 224.09662<br>179.03865 | 224.09644<br>179.03873 | 0.79<br>0.32 | – |
| $[M–2H+H]^+$ | $C_9H_{12}N_5S$ | 222.08102 | 222.08079 | 1.02 | Dehydrogenation |
| $[M–4H+H]^+$ | $C_9H_{10}N_5S$ | 220.06538 | 220.06514 | 1.08 | Dehydrogenation |
| $[M+O+H]^+$ | $C_9H_{14}N_5SO$ | 240.09145 | 240.09136 | 0.39 | Hydroxylation |
| $[M+O–2H+H]^+$ | $C_9H_{12}N_5SO$ | 238.07587 | 238.07571 | 0.68 | Hydroxylation/ Dehydrogenation |
| $[M+O–4H+H]^+$ | $C_9H_{10}N_5SO$ | 236.06026 | 236.06006 | 0.86 | Hydroxylation/ Dehydrogenation |
| $[M–S+O+H]^+$ | $C_9H_{14}N_5O$ | 208.11940 | 208.11929 | 0.55 | Desulfuration |
| $[M–S+O–2H+H]^+$ | $C_9H_{12}N_5O$ | 206.10374 | 206.10364 | 0.50 | Desulfuration/ Dehydrogenation |
| $[M–S+H]^+$ | $C_9H_{14}N_5$ | 192.12428 | 192.12437 | 0.48 | Desulfuration |
| $[M–S–2H+H]^+$ | $C_9H_{12}N_5$ | 190.10878 | 190.10872 | 0.31 | Desulfuration/ Dehydrogenation |
| $[M–CH_2+H]^+$ | $C_8H_{12}N_5S$ | 210.08044 | 210.08079 | 1.68 | Demethylation* |
| $[M–CH_2–2H+H]^+$ | $C_8H_{10}N_5S$ | 208.06487 | 208.06514 | 1.31 | Demethylation/ Dehydrogenation* |
| $[2M–2H+H]^+$ | $C_{18}H_{25}N_{10}S_2$ | 445.17007 | 445.16996 | 0.25 | Disulfide formation |
| $[2M–4H+H]^+$ | $C_{18}H_{23}N_{10}S_2$ | 443.15433 | 443.15431 | 0.05 | Disulfide formation/ Dehydrogenation |
| $[2M–6H+H]^+$ | $C_{18}H_{21}N_{10}S_2$ | 441.13870 | 441.13866 | 0.09 | Disulfide formation/ Dehydrogenation |

*observed after electrochemical oxidation only *via* LC-HRMS, but not via EC-HRMS

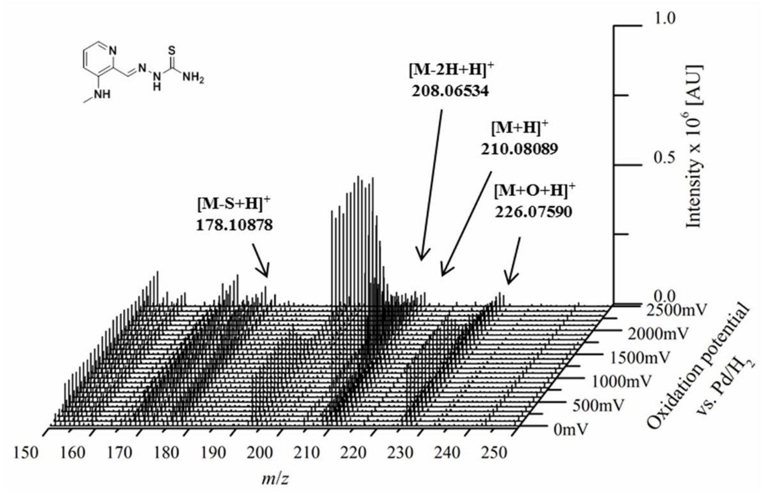


**Fig. S3** Mass voltammogram of thiosemicarbazone **3**

**Table S3** Metabolites of compound **3** detected by EC/MS

| **Modification** | **Sum formula** | **Detected *m/z*** | **Theoretical *m/z*** | **Rel. deviation [ppm]** | **Metabolic reaction** |
|---|---|---|---|---|---|
| $[M{+}H]^+$<br>$[M{-}NH_2]^+$ | $C_8H_{12}N_5S$<br>$C_8H_9N_4S$ | 210.08089<br>193.05434 | 210.08079<br>193.05424 | 0.46<br>0.50 | – |
| $[M{-}2H{+}H]^+$ | $C_8H_{10}N_5S$ | 208.06534 | 208.06514 | 0.05 | Dehydrogenation |
| $[M{-}4H{+}H]^+$ | $C_8H_8N_5S$ | 206.04982 | 206.04949 | 1.59 | Dehydrogenation |
| $[M{+}O{+}H]^+$ | $C_8H_{12}N_5SO$ | 226.07590 | 226.07571 | 0.85 | Hydroxylation |
| $[M{+}O{-}2H{+}H]^+$ | $C_8H_{10}N_5SO$ | 224.06024 | 224.06006 | 0.82 | Hydroxylation/ Dehydrogenation |
| $[M{+}O{-}4H{+}H]^+$ | $C_8H_8N_5SO$ | 222.04442 | 222.04441 | 0.06 | Hydroxylation/ Dehydrogenation |
| $[M{-}S{+}H]^+$ | $C_8H_{12}N_5$ | 178.10878 | 178.10872 | 0.33 | Desulfuration |
| $[M{-}S{-}2H{+}H]^+$ | $C_8H_{10}N_5$ | 176.09320 | 176.09307 | 0.73 | Desulfuration/ Dehydrogenation |
| $[M{-}S{-}4H{+}H]^+$ | $C_8H_8N_5$ | 174.07756 | 174.07742 | 0.79 | Desulfuration/ Dehydrogenation |
| $[M{-}CH_2{-}2H{+}H]^+$ | $C_7H_8N_5S$ | 194.04926 | 194.04949 | 1.20 | Demethylation/ Dehydrogenation* |
| $[2M{-}2H{+}H]^+$ | $C_{16}H_{21}N_{10}S_2$ | 417.13898 | 417.13866 | 0.77 | Disulfide formation |
| $[2M{-}4H{+}H]^+$ | $C_{16}H_{19}N_{10}S_2$ | 415.12317 | 415.12301 | 0.39 | Disulfide formation/ Dehydrogenation |
| $[2M{-}6H{+}H]^+$ | $C_{16}H_{17}N_{10}S_2$ | 413.10748 | 413.10736 | 0.29 | Disulfide formation/ Dehydrogenation |

*observed after electrochemical oxidation only *via* LC-HRMS, but not via EC-HRMS

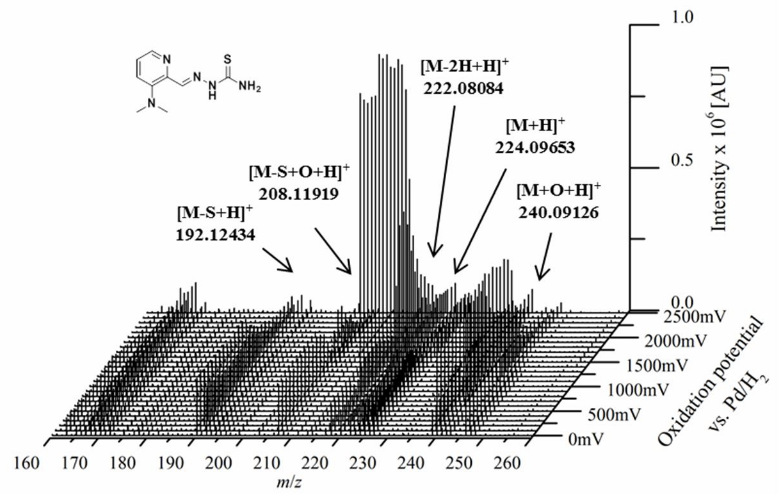


**Fig. S4** Mass voltammogram of thiosemicarbazone **4**

**Table S4** Metabolites of compound **4** detected by EC/MS

| **Modification** | **Sum formula** | **Detected *m*/*z*** | **Theoretical *m*/*z*** | **Rel. deviation [ppm]** | **Metabolic reaction** |
|---|---|---|---|---|---|
| $[M+H]^+$<br>$[M–NH_2]^+$ | $C_9H_{14}N_5S$<br>$C_9H_{11}N_4S$ | 224.09653<br>207.06984 | 224.09644<br>207.06989 | 0.39<br>0.26 | – |
| $[M–2H+H]^+$ | $C_9H_{12}N_5S$ | 222.08084 | 222.08079 | 0.21 | Dehydrogenation |
| $[M–4H+H]^+$ | $C_9H_{10}N_5S$ | 220.06526 | 220.06514 | 0.53 | Dehydrogenation |
| $[M+O+H]^+$ | $C_9H_{14}N_5SO$ | 240.09126 | 240.09136 | 0.41 | Hydroxylation |
| $[M+O–2H+H]^+$ | $C_9H_{12}N_5SO$ | 238.07567 | 238.07571 | 0.16 | Hydroxylation/ Dehydrogenation |
| $[M+O–4H+H]^+$ | $C_9H_{10}N_5SO$ | 236.06000 | 236.06006 | 0.24 | Hydroxylation/ Dehydrogenation |
| $[M–S+O+H]^+$ | $C_9H_{14}N_5O$ | 208.11919 | 208.11929 | 0.46 | Desulfuration |
| $[M–S+O–2H+H]^+$ | $C_9H_{12}N_5O$ | 206.10382 | 206.10364 | 0.89 | Desulfuration/ Dehydrogenation |
| $[M–S+H]^+$ | $C_9H_{14}N_5$ | 192.12434 | 192.12437 | 0.17 | Desulfuration |
| $[M–S–2H+H]^+$ | $C_9H_{12}N_5$ | 190.10870 | 190.10872 | 0.12 | Desulfuration/ Dehydrogenation |
| $[M–CH_2+H]^+$ | $C_8H_{12}N_5S$ | 210.08064 | 210.08079 | 0.73 | Demethylation |
| $[M–CH_2–2H+H]^+$ | $C_8H_{10}N_5S$ | 208.06523 | 208.06514 | 0.42 | Demethylation/ Dehydrogenation |
| $[M–CH_2–4H+H]^+$ | $C_8H_8N_5S$ | 206.04961 | 206.04949 | 0.57 | Demethylation/ Dehydrogenation |
| $[M–2xCH_2–2H+H]^+$ | $C_7H_8N_5S$ | 194.04929 | 194.04949 | 1.04 | Demethylation/ Dehydrogenation* |
| $[2M–2H+H]^+$ | $C_{18}H_{25}N_{10}S_2$ | 445.16980 | 445.16996 | 0.36 | Disulfide formation |
| $[2M–4H+H]^+$ | $C_{18}H_{23}N_{10}S_2$ | 443.15448 | 443.15431 | 0.39 | Disulfide formation/ Dehydrogenation |

*observed after electrochemical oxidation only *via* LC-HRMS, but not via EC-HRMS

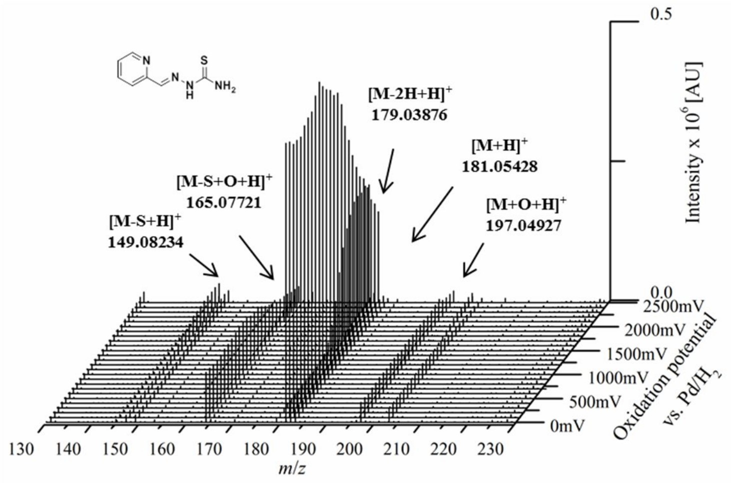


**Fig. S5** Mass voltammogram of thiosemicarbazone **6**

**Table S5** Metabolites of compound **6** detected by EC/MS

| Modification | Sum formula | Detected *m/z* | Theoretical *m/z* | Rel. deviation [ppm] | Metabolic reaction |
|---|---|---|---|---|---|
| $[M+H]^+$<br>$[M-NH_2]^+$ | $C_7H_9N_4S$<br>$C_7H_6N_3S$ | 181.05428<br>164.02782 | 181.05424<br>164.02769 | 0.20<br>0.77 | – |
| $[M-2H+H]^+$ | $C_7H_7N_4S$ | 179.03876 | 179.03859 | 0.93 | Dehydrogenation |
| $[M+O+H]^+$ | $C_7H_9N_4SO$ | 197.04927 | 197.04916 | 0.57 | Hydroxylation |
| $[M-S+O+H]^+$ | $C_7H_9N_4O$ | 165.07721 | 165.07709 | 0.74 | Desulfuration |
| $[M-S+O-2H+H]^+$ | $C_7H_7N_4O$ | 163.06158 | 163.06144 | 0.87 | Desulfuration/ Dehydrogenation |
| $[M-S+H]^+$ | $C_7H_9N_4$ | 149.08234 | 149.08217 | 1.12 | Desulfuration |
| $[M-S-2H+H]^+$ | $C_7H_7N_4$ | 147.06664 | 147.06652 | 0.80 | Desulfuration/ Dehydrogenation |
| $[2M-2H+H]^+$ | $C_{14}H_{15}N_8S_2$ | 359.08566 | 359.08556 | 0.28 | Disulfide formation |
| $[2M-4H+H]^+$ | $C_{14}H_{13}N_8S_2$ | 357.07013 | 357.06991 | 0.62 | Disulfide formation/ Dehydrogenation |

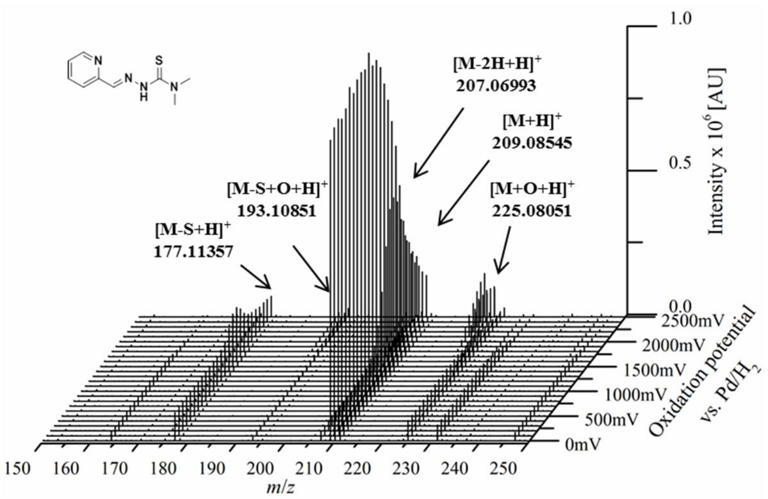


**Fig. S6** Mass voltammogram of thiosemicarbazone **8**

**Table S6** Metabolites of compound **8** detected by EC/MS

| Modification | Sum formula | Detected *m/z* | Theoretical *m/z* | Rel. deviation [ppm] | Metabolic reaction |
|---|---|---|---|---|---|
| $[M+H]^+$<br>$[M–N–2xCH_3]^+$ | $C_9H_{13}N_4S$<br>$C_7H_6N_3S$ | 209.08545<br>164.02774 | 209.08554<br>164.02769 | 0.49<br>0.28 | – |
| $[M–2H+H]^+$ | $C_9H_{11}N_4S$ | 207.06993 | 207.06989 | 0.18 | Dehydrogenation |
| $[M–4H+H]^+$ | $C_9H_9N_4S$ | 205.05449 | 205.05424 | 1.20 | Dehydrogenation |
| $[M+O+H]^+$ | $C_9H_{13}N_4SO$ | 225.08051 | 225.08046 | 0.23 | Hydroxylation |
| $[M+O–2H+H]^+$ | $C_9H_{11}N_4SO$ | 223.06491 | 223.06481 | 0.46 | Hydroxylation/ Dehydrogenation |
| $[M+O–4H+H]^+$ | $C_9H_9N_4SO$ | 221.04927 | 221.04916 | 0.51 | Hydroxylation/ Dehydrogenation |
| $[M–S+O+H]^+$ | $C_9H_{13}N_4O$ | 193.10851 | 193.10839 | 0.63 | Desulfuration |
| $[M–S+O–2H+H]^+$ | $C_9H_{11}N_4O$ | 191.09264 | 191.09274 | 0.51 | Desulfuration/ Dehydrogenation |
| $[M–S+H]^+$ | $C_9H_{13}N_4$ | 177.11357 | 177.11347 | 0.55 | Desulfuration |
| $[M–S–2H+H]^+$ | $C_9H_{11}N_4$ | 175.09785 | 175.09782 | 0.16 | Desulfuration/ Dehydrogenation |
| $[M–CH_2–2H+H]^+$ | $C_8H_9N_4S$ | 193.05426 | 193.05424 | 0.09 | Demethylation/ Dehydrogenation |
| $[M–2xCH_2–2H+H]^+$ | $C_7H_7N_4S$ | 179.03838 | 179.03859 | 1.19 | Demethylation/ Dehydrogenation* |
| $[2M–2H+H]^+$ | $C_{18}H_{23}N_8S_2$ | 415,14828 | 415.14816 | 0.29 | Disulfide formation |
| $[2M–4H+H]^+$ | $C_{18}H_{21}N_8S_2$ | 413.13266 | 413.13251 | 0.36 | Disulfide formation/ Dehydrogenation |

*observed after electrochemical oxidation only *via* LC-HRMS, but not via EC-HRMS

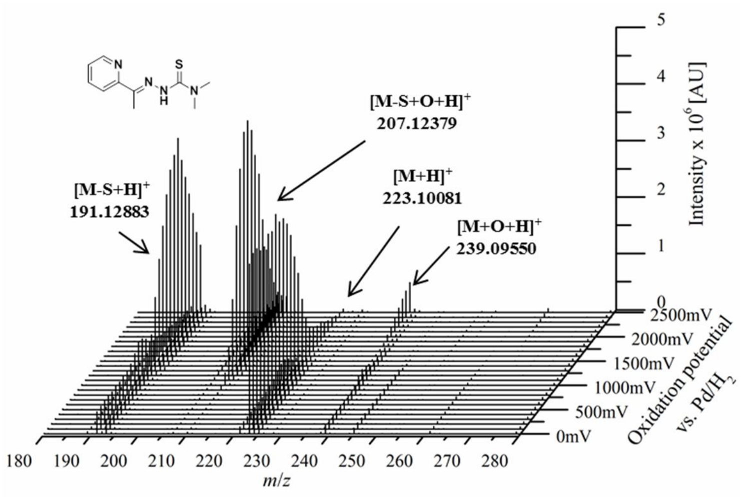


**Fig. S7** Mass voltammogram of thiosemicarbazone **9**

**Table S7** Metabolites of compound **9** detected by EC/MS

| Modification | Sum formula | Detected *m*/*z* | Theoretical *m*/*z* | Rel. deviation [ppm] | Metabolic reaction |
|---|---|---|---|---|---|
| $[M+H]^+$<br>$[M-N-2xCH_3]^+$ | $C_{10}H_{15}N_4S$<br>$C_8H_8N_3S$ | 223.10081<br>178.04292 | 223.10119<br>178.04334 | 1.72<br>2.39 | – |
| $[M+O+H]^+$ | $C_{10}H_{15}N_4SO$ | 239.09550 | 239.09611 | 2.54 | Hydroxylation |
| $[M+O-2H+H]^+$ | $C_{10}H_{13}N_4SO$ | 237.07994 | 237.08046 | 2.19 | Hydroxylation/ Dehydrogenation |
| $[M-S+O+H]^+$ | $C_{10}H_{15}N_4O$ | 207.12379 | 207.12404 | 1.20 | Desulfuration |
| $[M-S+O-2H+H]^+$ | $C_{10}H_{13}N_4O$ | 205.10796 | 205.10839 | 2.09 | Desulfuration/ Dehydrogenation |
| $[M-S+H]^+$ | $C_{10}H_{15}N_4$ | 191.12883 | 191.12912 | 1.53 | Desulfuration |
| $[M-S-2H+H]^+$ | $C_{10}H_{13}N_4$ | 189.11314 | 189.11347 | 1.76 | Desulfuration/ Dehydrogenation |
| $[M-CH_2-2H+H]^+$ | $C_9H_{11}N_4S$ | 207.06976 | 207.06989 | 0.65 | Demethylation/ Dehydrogenation* |
| $[M-2xCH_2-2H+H]^+$ | $C_8H_9N_4S$ | 193.05423 | 193.05424 | 0.07 | Demethylation/ Dehydrogenation* |
| $[M-3xCH_2-2H+H]^+$ | $C_7H_7N_4S$ | 179.03842 | 179.03859 | 0.97 | Demethylation/ Dehydrogenation* |
| $[2M-2H+H]^+$ | $C_{20}H_{25}N_8S_2$ | 441.16302 | 441.16381 | 1.79 | Disulfide formation |
| $[2M-4H+H]^+$ | $C_{20}H_{23}N_8S_2$ | 439.14792 | 439.14816 | 0.55 | Disulfide formation/ Dehydrogenation |

*observed after electrochemical oxidation only *via* LC-HRMS, but not via EC-HRMS

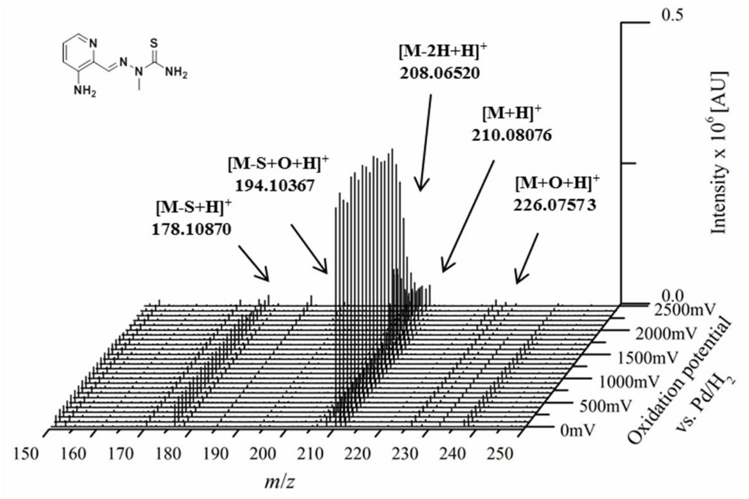


**Fig. S8** Mass voltammogram of thiosemicarbazone **10**

**Table S8** Metabolites of compound **10** detected by EC/MS

| **Modification** | **Sum formula** | **Detected *m*/*z*** | **Theoretical *m*/*z*** | **Rel. deviation [ppm]** | **Metabolic reaction** |
|---|---|---|---|---|---|
| $[M+H]^+$ | $C_8H_{12}N_5S$ | 210.08076 | 210.08079 | 0.16 | – |
| $[M–2H+H]^+$ | $C_8H_{10}N_5S$ | 208.06520 | 208.06514 | 0.28 | Dehydrogenation |
| $[M–4H+H]^+$ | $C_8H_8N_5S$ | 206.04945 | 206.04949 | 0.21 | Dehydrogenation |
| $[M+O+H]^+$ | $C_8H_{12}N_5SO$ | 226.07573 | 226.07571 | 0.10 | Hydroxylation |
| $[M+O–2H+H]^+$ | $C_8H_{10}N_5SO$ | 224.06000 | 224.06006 | 0.26 | Hydroxylation/ Dehydrogenation |
| $[M+O–4H+H]^+$ | $C_8H_8N_5SO$ | 222.04443 | 222.04441 | 0.10 | Hydroxylation/ Dehydrogenation |
| $[M–S+O+H]^+$ | $C_8H_{12}N_5O$ | 194.10367 | 194.10364 | 0.17 | Desulfuration |
| $[M–S+O–2H+H]^+$ | $C_8H_{10}N_5O$ | 192.08794 | 192.08799 | 0.24 | Desulfuration/ Dehydrogenation |
| $[M–S+H]^+$ | $C_8H_{12}N_5$ | 178.10870 | 178.10872 | 0.12 | Desulfuration |
| $[M–S–2H+H]^+$ | $C_8H_{10}N_5$ | 176.09303 | 176.09307 | 0.24 | Desulfuration/ Dehydrogenation |
| $[M–S–4H+H]^+$ | $C_8H_8N_5$ | 174.07747 | 174.07742 | 0.28 | Desulfuration/ Dehydrogenation |
| $[2M–2H+H]^+$ | $C_{16}H_{21}N_{10}S_2$ | 417.13843 | 417.13866 | 0.55 | Disulfide formation |
| $[2M–4H+H]^+$ | $C_{16}H_{19}N_{10}S_2$ | 415.12292 | 415.12301 | 0.21 | Disulfide formation/ Dehydrogenation |

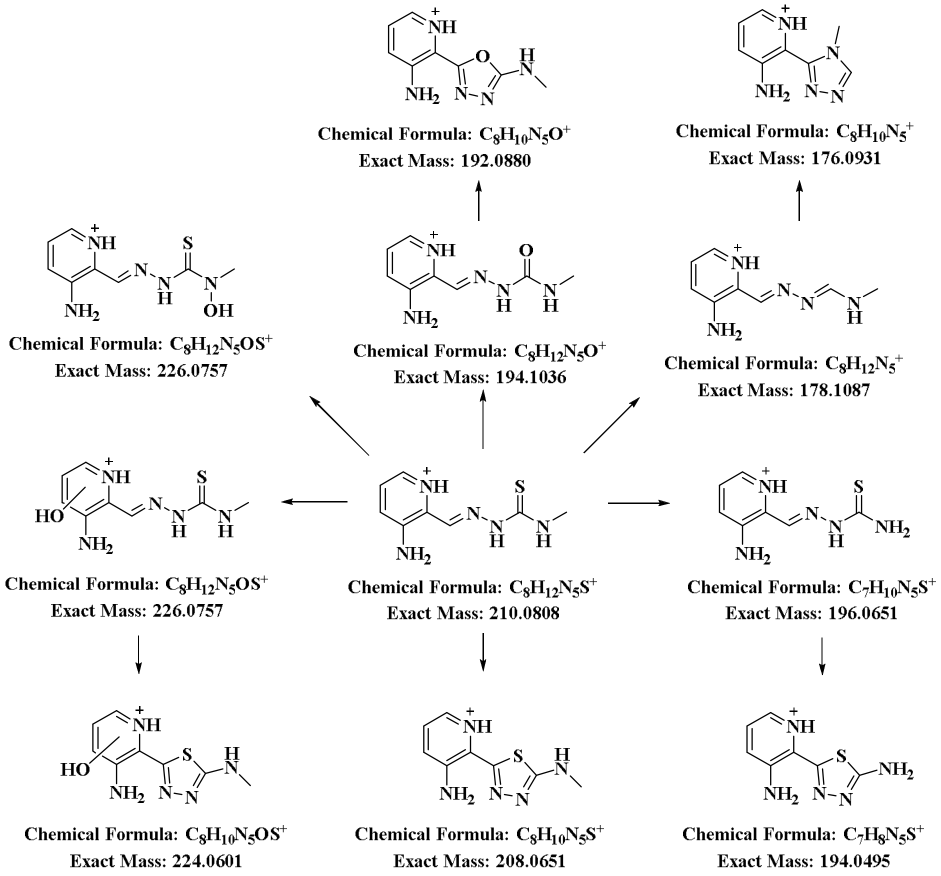


**Scheme S2** Metabolic pathways of **1** elucidated by LC-HRMS after incubation with cell-free microsomes

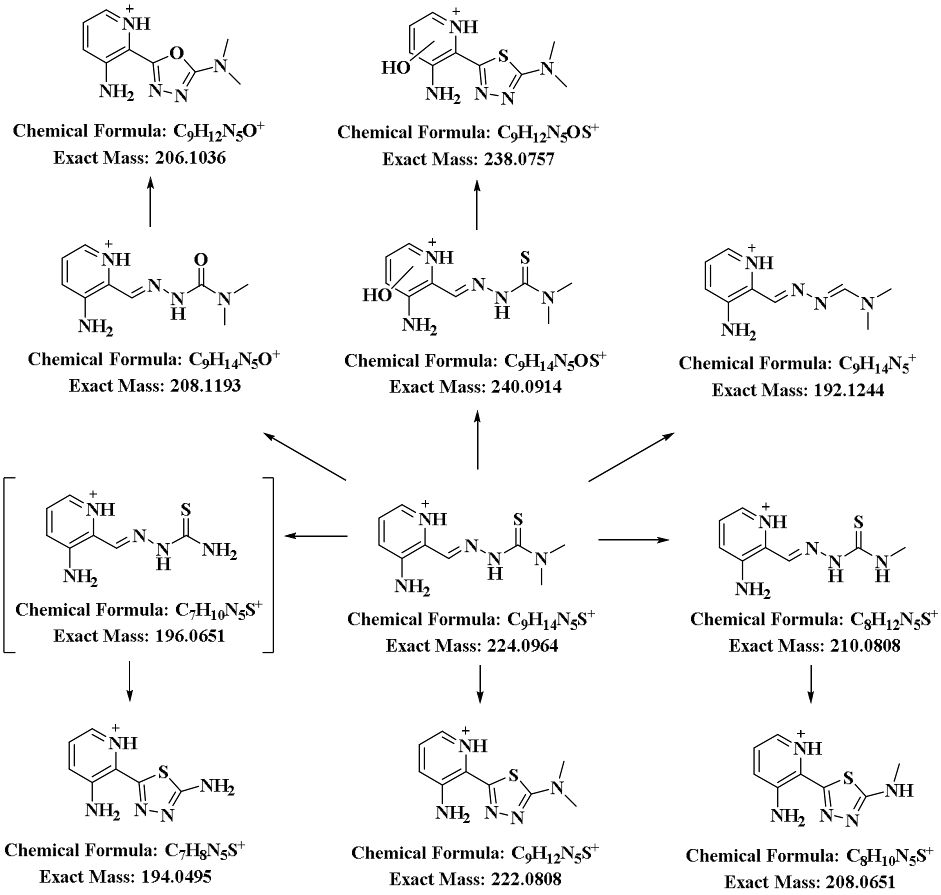


**Scheme S3** Metabolic pathways of **2** elucidated by LC-HRMS after incubation with cell-free microsomes

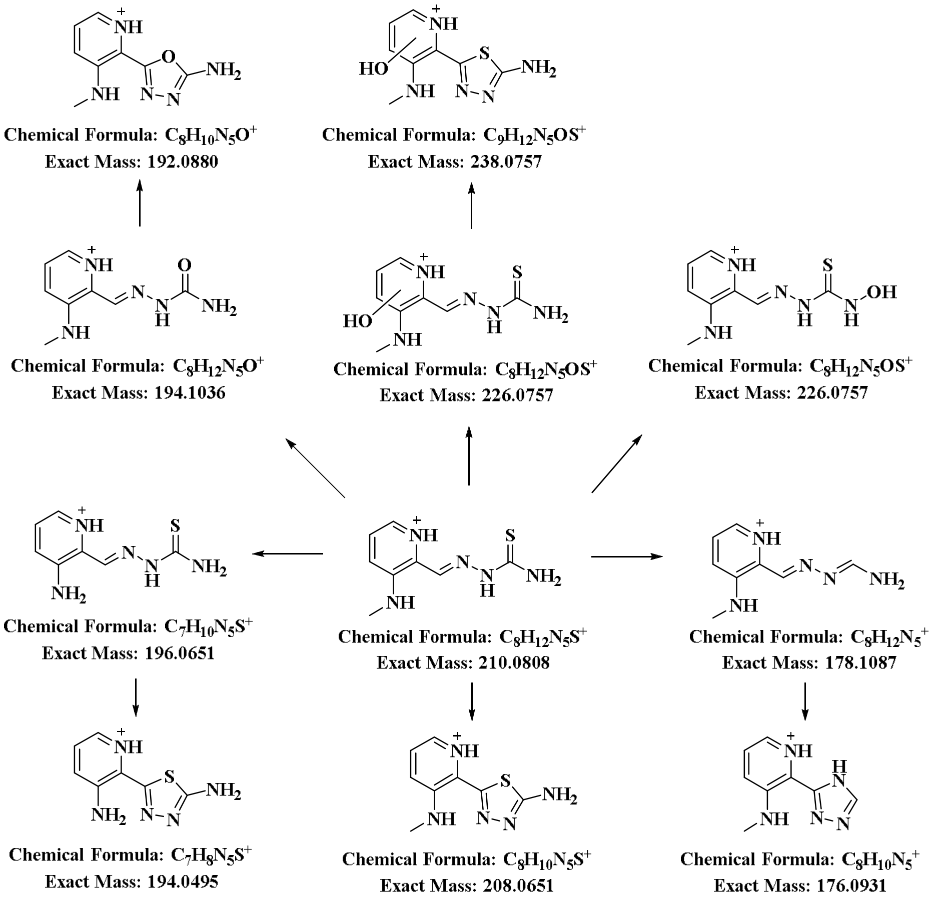


**Scheme S4** Metabolic pathways of **3** elucidated by LC-HRMS after incubation with cell-free microsomes

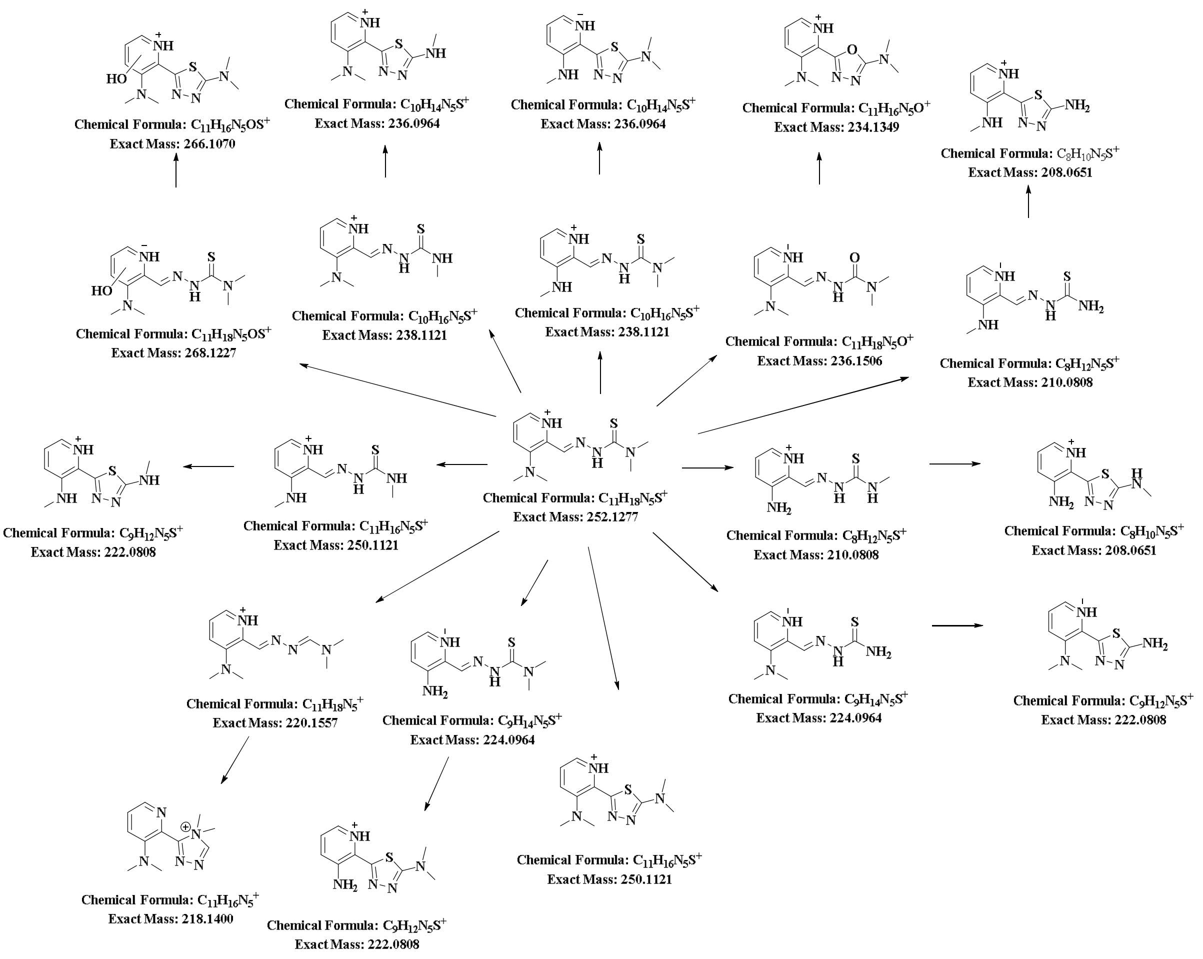


**Scheme S5** Metabolic pathways of **5** elucidated by LC-HRMS after incubation with cell-free microsomes

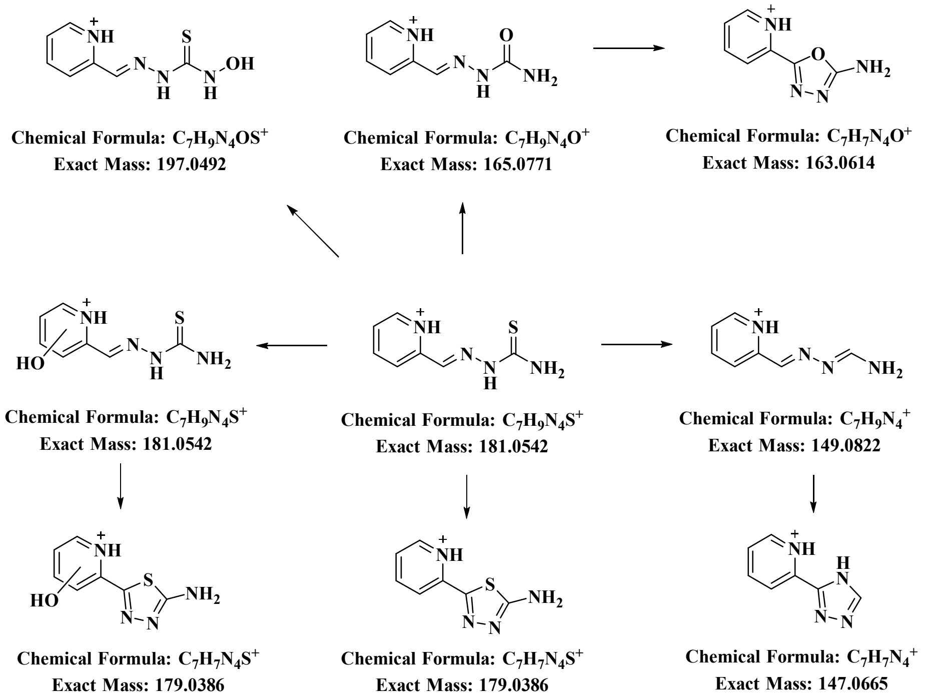


**Scheme S6** Metabolic pathways of **6** elucidated by LC-HRMS after incubation with cell-free microsomes

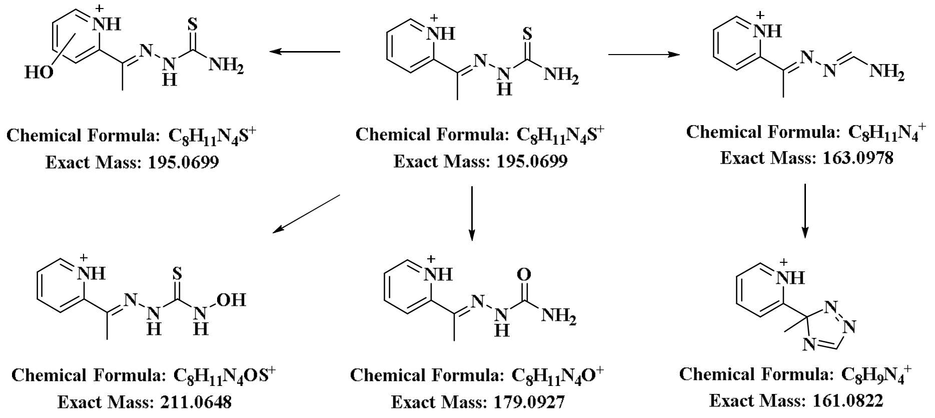


**Scheme S7** Metabolic pathways of **7** elucidated by LC-HRMS after incubation with cell-free microsomes

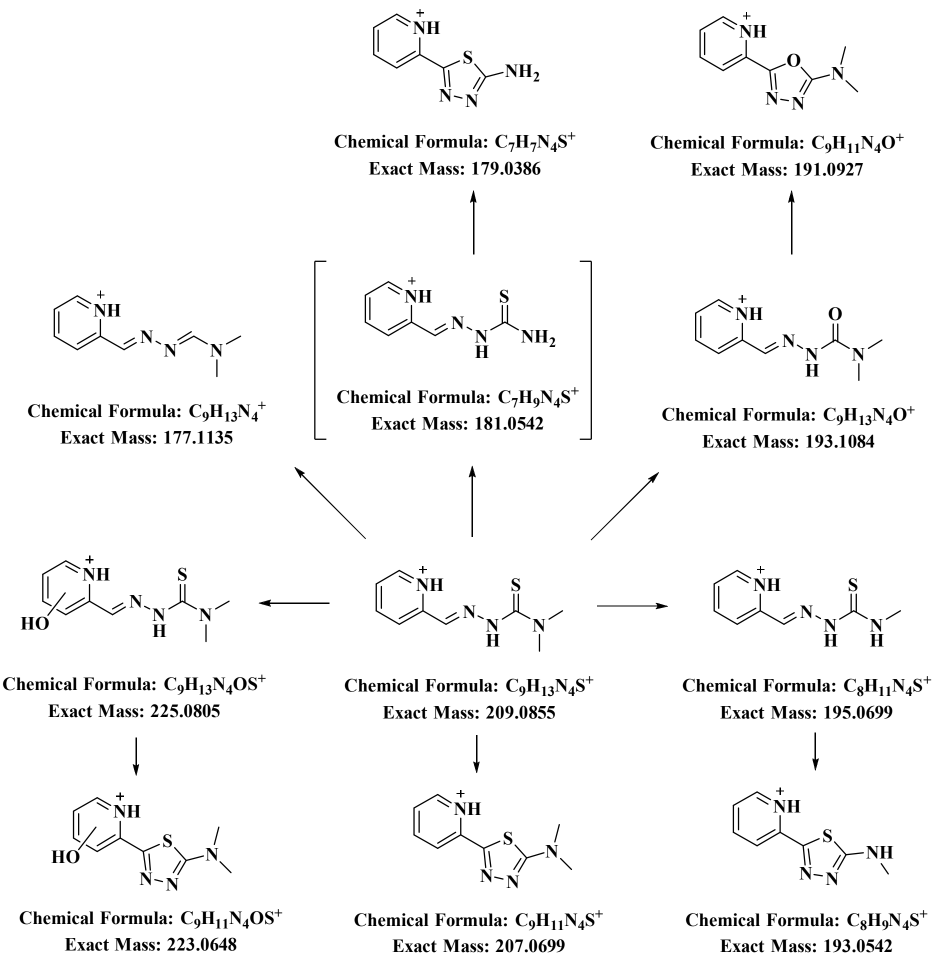


**Scheme S8** Metabolic pathways of **8** elucidated by LC-HRMS after incubation with cell-free microsomes

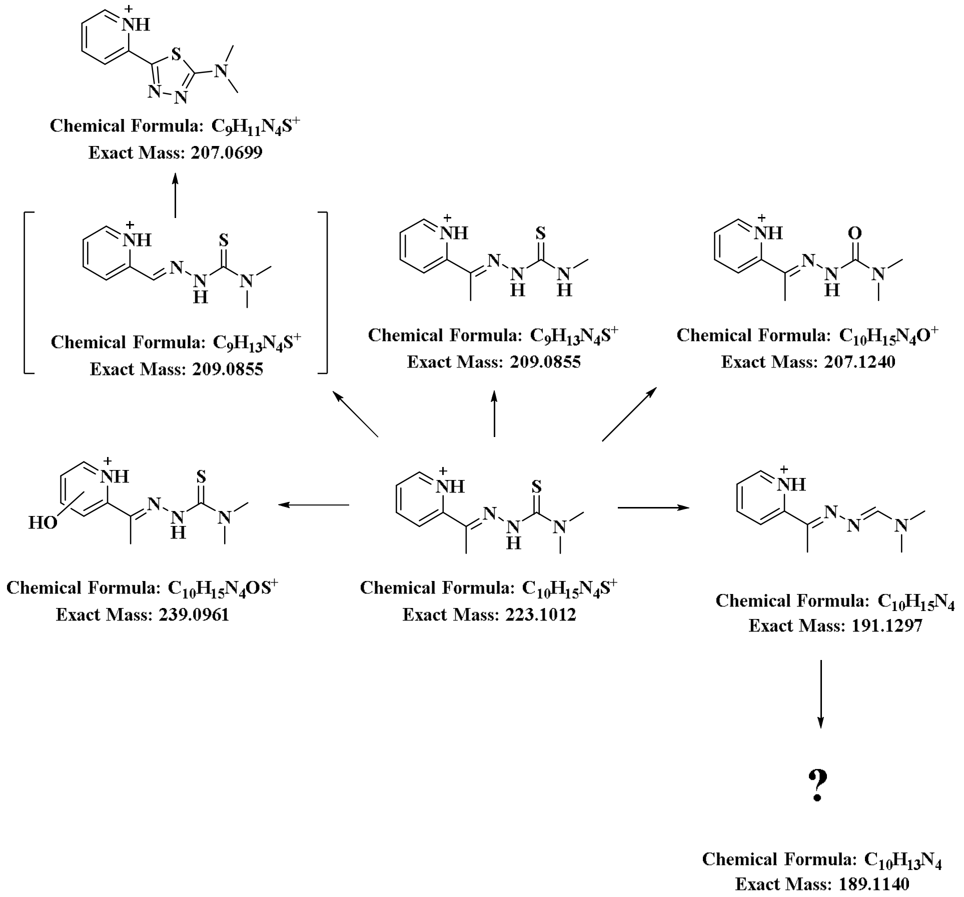


**Scheme S9** Metabolic pathways of **9** elucidated by LC-HRMS after incubation with cell-free microsomes

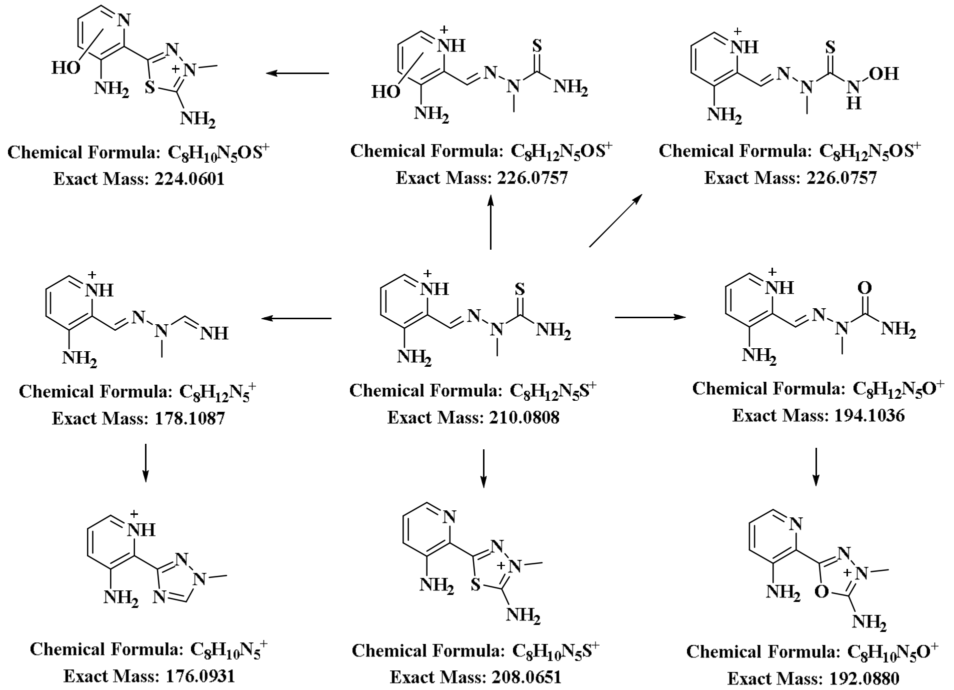


**Scheme S10** Metabolic pathways of **10** elucidated by LC-HRMS after incubation with cell-free microsomes

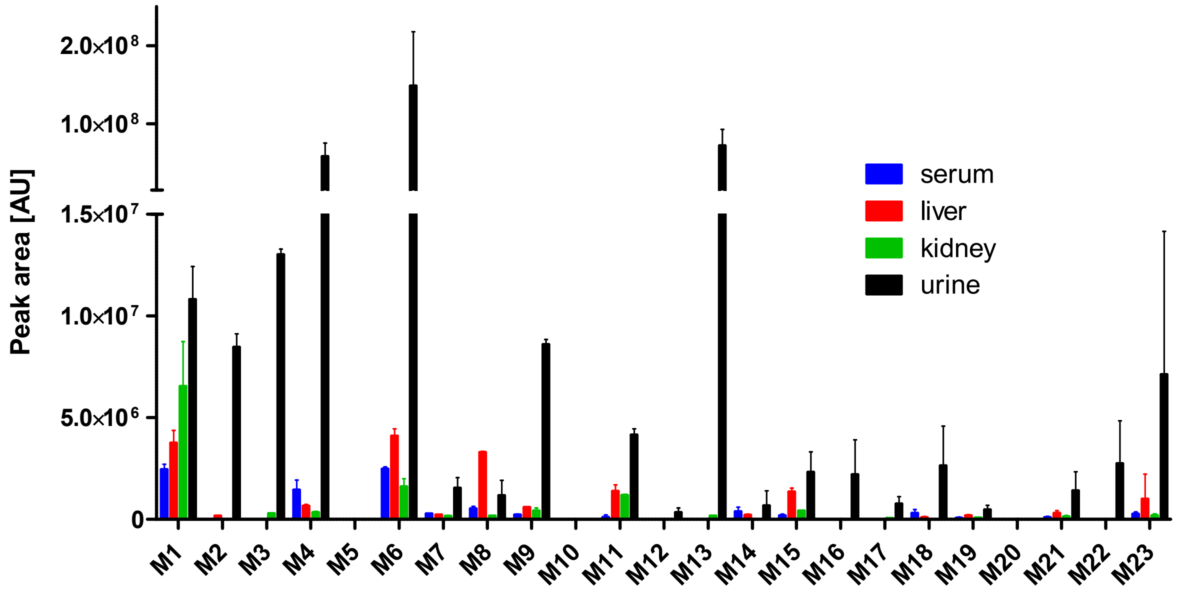


**Fig. S9** Relative quantification of metabolites 1–23 of compound **5** in serum/liver/kidney/urine samples of mice (zoom of the data of Figure 8)
